# Research on Intelligent Thermal Optimization for Chiplet-Based Heterogeneously Integrated AI Chip Embedded with Leaf-Vein-Inspired Fractal Microchannels

**DOI:** 10.3390/ma19040679

**Published:** 2026-02-10

**Authors:** Jie Wu, Yu Liang, Guibin Liu, Ruiyang Pang, Yi Teng, Chen Li, Xuetian Bao, Shi Lei, Zhikuang Cai

**Affiliations:** 1 College of Integrated Circuit Science and Engineering (College of Industry-Education Integration), Nanjing University of Posts and Telecommunications, Nanjing 210003, China; 1323229333@njupt.edu.cn (Y.L.); 1224228506@njupt.edu.cn (G.L.); 1223228215@njupt.edu.cn (R.P.); 1323229334@njupt.edu.cn (Y.T.); whczk@njupt.edu.cn (Z.C.); 2 Tongfu Microelectronics Co., Ltd., Nantong 226004, China; li.chen@tfme.com (C.L.); bao.xuetian@tfme.com (X.B.); shi.lei@tfme.com (S.L.)

**Keywords:** fractal microchannel, heterogeneous integration, machine learning, multi-parameter optimization, heat dissipation

## Abstract

Conventional cooling schemes that rely on rigid heat-sink-to-die coupling in vertical stacks fail to track the dynamic, non-uniform heat map of high-performance artificial-intelligence (AI) chips employing chiplet-based heterogeneous integration, giving rise to local hot spots. To eliminate this mismatch, we present a leaf-vein-inspired fractal microchannel tailored for such AI processors. Its hierarchical bifurcation–confluence topology adaptively reshapes the flow field, delivering ultra-low thermal resistance, high heat-transfer coefficients, and uniform dissipation. Coupled with reconfigurable chiplet placement, the design is evaluated through FEM-based orthogonal experiments that rank the influence of coolant, channel diameter/depth, inlet/outlet position, substrate thickness, and flow rate via range analysis and Analysis of Variance (ANOVA). A machine-learned surrogate model of junction temperature is then fed to Particle Swarm Optimization (PSO) for multi-parameter optimization. When re-simulated with the optimal parameter set, the symmetric fractal network lowered the AI chip junction temperature from 127.80 °C to 30.97 °C, a 76% improvement, offering a theoretical basis for hotspot mitigation in advanced heterogeneous AI packages.

## 1. Introduction

Driven by the relentless demand for extreme performance and seamless integration in modern electronics, chiplet-based heterogeneous integration has emerged as a key enabler for next-generation AI processors [1,2]. By leveraging advanced packaging to integrate chiplets of disparate materials, process nodes, and functionalities, this technology lets AI chips deliver unprecedented computational power within ever-shrinking footprints, meeting the exacting requirements of AI, big-data analytics, cloud computing, and beyond. However, the prodigious heat generated during operation threatens both performance and reliability. Studies have indicated [3,4,5] that temperature-induced failures account for up to 55% of failures under various service conditions, and for every 10 °C increase in chip temperature, the failure probability increases by an order of magnitude. Consequently, efficient thermal management stands as the foremost challenge in the chiplet-based heterogeneous integration of AI chips.

Interlayer microchannel cooling technology, renowned for its high efficiency and flexibility, has emerged as a key solution to address this thermal challenge. By leveraging the high specific surface area provided by microchannel structures, this technology ensures thorough contact between the coolant and the chip surface, enabling rapid and uniform heat dissipation. It not only effectively reduces chip temperature but also guarantees stability and reliability under high-power operation. Existing research [6,7,8,9,10,11,12,13,14] has systematically optimized key parameters from topological, geometric, and flow perspectives. Brunschwiler [6] demonstrated that double-sided interlayer cooling can suppress thermal gradients in 3D-ICs to <50 K, making it the only viable solution for multi-chip stacking. Xia [10] found that fan-shaped cavities significantly weaken boundary layer stagnation compared to triangular cavities. S Ndao [11] and Wan [12] confirmed that circular turbulators/pin-fins exhibit optimal overall performance under identical pumping power, though excessively low pitch and porosity drastically increase pumping requirements. Ditri [13] integrated micro-pin-fin arrays (μPFAs) into GaN-on-SiC, reducing junction-to-coolant thermal resistance to 0.12 K·cm^2^/W. Wei [14] employed an LPBF-fabricated variable-aperture (80–300 μm) nozzle array, achieving 0.03 K·cm^2^/W thermal resistance at 1000 W/cm^2^, with a heat transfer coefficient of 172 kW/m^2^·K and a pressure drop below 35 kPa. These studies collectively revealed that the performance ceiling of conventional microchannels is set by the intricate synergy among geometry, flow, and topology; once these freedom degrees are exhausted, further gains become asymptotically small. Breaking this plateau requires a paradigm shift from incremental parameter tuning to architectural innovation. Bio-inspired microchannels, which transplant hierarchical, adaptive, and multi-scale designs found in nature into engineered cooling manifolds, have recently emerged as such a solution. Yue et al. [15] utilized a vascular honeycomb composite structure to create multi-stage diverging/converging channels, enhancing coolant mixing and boosting heat transfer efficiency by 64.5%. Gao et al. [16] designed a converging–diverging honeycomb (CM-H) microchannel inspired by goosefish ribs, compressing high-temperature zones near the ribs through tapered cross-sections and significantly improving temperature uniformity. He et al. [17] performed multi-objective optimization on a Y-shaped fractal manifold, where a symmetric structure achieved lower temperature differences and more uniform thermal fields at identical fractal levels. These findings reveal that coupling symmetric fractal topologies with multi-stage diverging–converging flow can precisely embed high-temperature zones within the core flow region, simultaneously expanding the heat transfer area while balancing temperature uniformity and low power consumption. This mechanistic consensus provides direct and critical inspiration for the Leaf Vein-like Bionic Fractal Microchannel Manifold Structure (LLVBF-MMS) proposed in this work. Furthermore, the research also indicates that microchannel geometry, dimensions, layout, and integration strategies critically determine cooling performance. However, the complex coupling of these parameters makes manual co-optimization time-consuming and imprecise. Consequently, developing intelligent co-optimization strategies is not only an urgent necessity to overcome the limitations of manual trial-and-error but also a key opportunity to fully leverage the integrated advantages of LVBF-MMS in multi-stage flow distribution, uniform temperature control, and low power consumption.

Intelligent optimization algorithms offer a powerful tool to address this co-optimization challenge. These methods enable intelligent parameter optimization of microchannel structures, achieving performance balance under multiple constraints. Weighted real-coded genetic algorithms, for instance, optimize design parameters within specified dimensional ranges [18,19,20]. Hwang et al. [21] combined a multi-objective genetic algorithm with 3D conjugate heat transfer simulation to optimize asymmetric converging–diverging channels, identifying a Pareto-optimal configuration (8-degree convergence angle, 1:2.5 expansion ratio) under a pressure drop constraint (<150 kPa), which enhanced the heat transfer coefficient by 19.8 times. Gurpinar et al. [22] integrated Fourier series-based thermal modeling (computational speed 47 times faster than FEM) with the NSGA-III algorithm to simultaneously optimize thermal resistance, weight, and flow resistance at 500 W/cm^2^. The optimized fractal pin-fin array increased turbulent intensity by 2.8 times while reducing material volume by 31.5%. Chen et al. [23] proposed a deep reinforcement learning (DRL) framework coupled with 3D conjugate heat transfer to optimize grooved microchannels at 500 W/cm^2^. The optimal configuration (1.8 aspect ratio, 2.5 groove width ratio) achieved thermal resistance of 0.07 K·cm^2^/W (31% improvement) and a pressure drop of 12.3 kPa (42% improvement). These methods dynamically optimize microchannels according to chip requirements, reducing energy consumption while ensuring performance and reliability. Nevertheless, existing research exhibits a significant gap in utilizing intelligent algorithms, particularly neural network-based multi-parameter optimization, to predict and optimize junction temperatures for reconfigurable heat-source chips, limiting their applicability in tackling the dynamic thermal management challenges of heterogeneously integrated chiplets.

To tackle the thermal challenges of AI chips, we propose an intelligent thermal optimization methodology based on leaf-vein-inspired fractal microchannels, synergistically implemented through chiplet-based heterogeneous integration that inherently supports heat-source reconfiguration. High-power chiplets dynamically migrate to the coolest zones of the fractal manifold as workloads and power maps evolve. Orthogonal experiments screen dominant geometric parameters, whose impact on junction temperature is quantified via finite-element models that concurrently consider coolant type and flow rate. Key parameters span coolant medium and flow velocity, microchannel diameter, depth, inlet/outlet positions, and substrate thickness. Range and variance analyses rank factor significance, after which a machine-learning-driven multi-parameter optimizer jointly refines coolant selection, flow conditions, and channel geometry. Thermal simulations of the reconfigured chiplet-microchannel stack validate synergistic gains in cooling efficiency. Currently, the reconfigurable optimization simulation of heat source positions is conducted under static heat loads, and due to space limitations of the paper, the study on dynamic feasibility is not included at present. In future work, we will further simulate and experimentally verify the results of dynamic reconfigurable heat dissipation optimization. This AI-orchestrated co-optimization framework, validated on a reconfigurable chiplet architecture, provides actionable design guidance for next-generation AI accelerators targeting energy-efficient, high-density computing platforms.

## 2. Experiment and Methodology

### 2.1. Model Construction with Chiplet-Based Heterogeneous Integration

**Figure 1:**
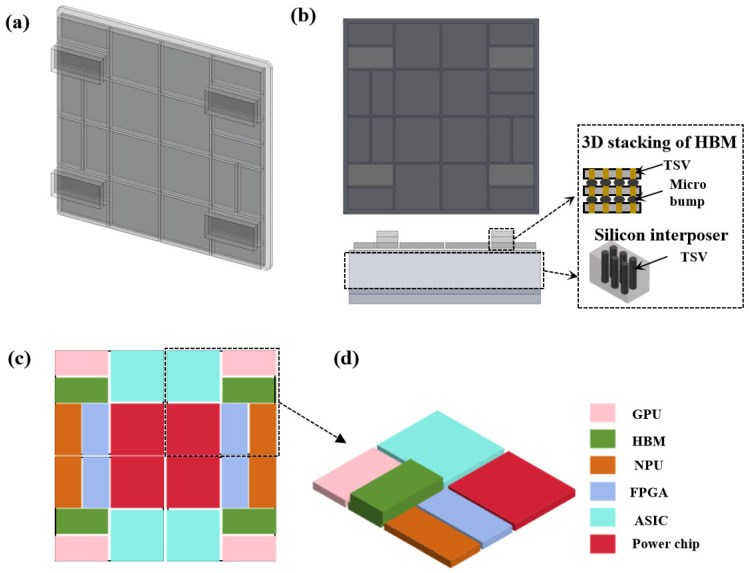
Structural model of chiplet heterogeneous integration. (**a**) Three-dimensional front view, (**b**) main view and side view, (**c**) chiplet arrangement, (**d**) individual components of a single chiplet.

**Figure 2:**
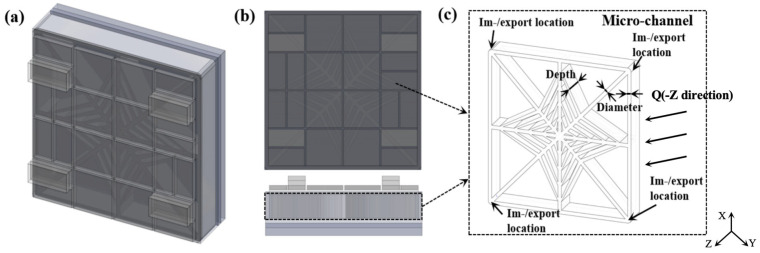
Schematic diagram of the embedded leaf vein bionic fractal microchannel manifold structure. (**a**) Three-dimensional front view, (**b**) main view and side view, (**c**) microchannel framework diagram.

First, 3D models of individual chiplets were created using the computer-aided design software SolidWorks (SOLIDWORKS 2024), as illustrated in Figure 1. AI chips achieve heterogeneous computing power integration and system-level performance leap by heterogeneously integrating chiplets with distinct functionalities. These chiplets include a GPU, FPGA, NPU, ASIC, power modules, HBM, etc., each undertaking specific computational tasks within the AI chip [24,25]. The GPU (graphics processing unit) handles graphics and parallel computing tasks; the FPGA (field-programmable gate array) offers reconfigurability to flexibly adapt to diverse computational demands; the NPU (neural processing unit) is optimized for neural network computations to deliver high-efficiency processing capabilities; and the ASIC (application-specific integrated circuit), excels in specific algorithms with high efficiency, significantly reducing power consumption. Figure 1b illustrates the side view where TSVs are embedded within the silicon interposer. The AI chip with heterogeneous chiplet integration is bonded to the silicon interposer using microbumps, while solder ball connections are employed between the interposer and the package substrate. The HBM stacked chips are interconnected using TSVs and microbumps. The HBM module employs 3D stacking technology (Figure 1b), providing high memory bandwidth to meet the AI chip’s requirements for high-speed data read/write operations [14]. The heat generated by the power module elevates the liquid temperature at the microchannel inlet and affects heat dissipation in adjacent AI core regions. It is essential to ensure that the microchannels efficiently dissipate heat from both the AI computing cores and the power module simultaneously. A co-design approach with reconfigurable heat sources ensures that both the power module and the heterogeneously integrated AI chip operate within safe temperature ranges, mitigating reliability issues caused by thermal stress. Heterogeneous integration primarily achieves high-density interconnects between chiplets through silicon interposers and TSV (Through-Silicon Via) technology (Figure 2b) [26]. Detailed parameters of the chiplets and interconnects are listed in Table 1.

The microchannel is embedded in the center of the silicon interposer, directly below the heat source. The heat generated by the chip is conducted through the solder joints to the silicon interposer (integrated with microchannels) via thermal conduction and subsequently removed from the package by the circulating microfluid. In the Z-direction of the equivalent model, a heat flux (Q) enters the model from its upper surface, creating a temperature difference (ΔT) between the upper and lower surfaces. The entire heat flux (Q) exits exclusively from the lower surface of the model.

The thermal resistance network model is analogous to an electrical resistance diagram (Figure S5), where thermal resistance is still defined only along the direction of heat propagation (A → D), and heat flow strictly adheres to the second law of thermodynamics (from high-temperature points to low-temperature points).

### 2.2. Co-Design Methodology and Thermal Analysis of Embedded LVBF-MMS

#### 2.2.1. Design of Embedded Leaf-Vein Bionic Fractal Microchannel Manifold Structure

Currently, the structural design of biomimetic fractal heat transfer structures predominantly adopts Murray’s law and its derived parameters. In this paper, the design of an embedded leaf-vein-inspired fractal microchannel manifold structure features arc-shaped inlets and outlets instead of right-angled ones. As the coolant flows out from the annular area, regions farther from the outlet require more time to discharge, allowing the coolant to promptly dissipate heat generated by the heat source. This results in localized temperature reduction near the microchannel inlets and outlets. Additionally, designing a Y-shaped flow channel to converge the manifold branches closest to the power chip not only reduces the temperature of the heat source but also improves its temperature distribution. In the preliminary stage, four types of microchannel structures were designed (Figure S2), including circular, octagonal, quadrangular, and star-shaped leaf-vein bionic designs. Under identical channel diameters and a flow velocity of 0.5 m/s, the circular chip exhibited the lowest junction temperature of 27.57 °C; the octagonal chip reached 27.51 °C; the quadrangular chip achieved 27.46 °C; and the star-shaped chip recorded the lowest junction temperature of 27.33 °C. According to the convective heat flux equation (q), the fluid cross-sectional areas were 64.9095 mm^2^ for the circular type, 69.0112 mm^2^ for the octagonal type, 42.9466 mm^2^ for the quadrangular type, and 27.0000 mm^2^ for the star-shaped type. A smaller fluid cross-sectional area ($A_{s}$) corresponds to a higher heat flux and superior heat dissipation. This study adopted the star-shaped leaf-vein bionic design, which demonstrated the optimal heat dissipation effect based on chip junction temperature.

The final stellar configuration was derived from the bionic blade geometric structure through a four-step process grounded in evolutionary efficiency: (1) Inspired by the leaf vascular system’s million-year-refined Pareto optimum among transport distance, pressure drop, and heat dissipation area, Murray’s law quantifies the bifurcation diameter ratio as n^(−1/3)^ to directly map microchannel diameter-length relationships, thereby eliminating the need for exhaustive topological searches. (2) The leaf-vein network surpasses conventional straight channels by offering enhanced local Nusselt numbers through increased bifurcation points, reduced maximum temperature differences via multi-path center-to-edge flow distribution, and simplified neural network surrogate model training through fractal-based parametric description. (3) The star-leaf-vein structure retains a three-level branching topology (primary → secondary → tertiary mesh veins) with diameters proportionally scaled from 1 mm to 0.7 mm to 0.5 mm, with each bifurcation verified via Murray’s law to ensure minimal pumping power. (4) Four candidates with equivalent flow area were generated within a 60 × 60 mm cold plate—circular, octagonal, quadrangular, and star-shaped (Figure S2)—and rapid CFD scoring under fixed conditions (0.5 m/s flow velocity, 130 W heat source) proved the star-shaped cross-section to be optimal, exhibiting the smallest area (27 mm^2^) and lowest junction temperature (27.33 °C), thus establishing it as a baseline for subsequent optimization.

#### 2.2.2. Thermal Analysis of Embedded Leaf-Vein-Inspired Bionic Fractal Microchannel Manifold Structures

The microchannel architecture in this work is based on a LVBF-MMS. A three-dimensional solid model (Figure 2a) was created in SolidWorks. Key design parameters, including the working fluid, channel diameter and depth, inlet/outlet locations, substrate thickness, and flow velocity, are summarized in Figure 2c. To minimize corner losses, 90° bends are blended with continuous arcs, yielding an arc–straight hybrid geometry that lengthens the flow-guiding region, suppresses centrifugal deviation, and eliminates low-velocity vortices near walls, thereby enhancing velocity symmetry. A silicon interposer fabricated via standard Si processes and equipped with TSVs provides high-density electrical routing and efficient heat removal. Near-junction cooling is further employed to maximize thermal performance. The simulation parameters are listed in Table 2.

The constructed microchannel model was imported into the fluid flow module of Ansys Fluent software (ANSYS2023). Detailed simulation parameters are presented in Table 3. Within Ansys, the fluid region (representing the coolant flow path within the microchannels) and the solid region (comprising the microchannel walls, chip substrate, and other solid structures) were defined, respectively. Polyhedral meshing was employed to generate high-quality grids to better capture fluid flow and heat transfer details, thereby enhancing simulation accuracy. The computational domain featured a hybrid mesh: the global structure comprised 1,531,022 polyhedral cells with inflation layers near walls; 1,631,274 boundary nodes, 392,953 boundary faces, and 127 boundary face zones. The boundary layer resolution consisted of 3 layers with a growth ratio of 1.2. Key refinements included localized hexahedral meshing at the fractal bifurcation of leaf veins (minimum dimension of 0.1 mm) and the peripheral boundary regions of microfluidic channels. The final mesh balanced accuracy (grid convergence index < 1.5%) and computational efficiency. Studies have shown that the results of device temperature fields are independent of mesh quality, thus eliminating the need for grid dependency analysis. A visualization of the mesh is provided in Figure S3. For boundary conditions, the inlet was set as a velocity inlet to simulate coolant entering the microchannels at a specified velocity. The outlet was set as pressure outlet, with the outlet pressure set to 0, representing free outflow of the coolant. Walls were defined as no-slip boundaries. The simulation specified an inlet water temperature of 26.5 °C. A heat flux of 130 W (36,111 W/m^2^) was applied to the bottom surface of the heat source to simulate heat generated during operation. The SIMPLE algorithm couples the momentum–energy equations; a 600 s transient run (1000 iterations, starting from the inlet) guaranteed convergence and accuracy. The total cross-sectional area of the heat source was As = 60 mm × 60 mm, with total power loss of P_loss_ = 130 W. The average heat flux density of the Si-based chip heat source was calculated as

q_loss_ = P_loss_·As = 1300.06 × 0.06 = 36,111 W/m^2^ (1)

A constant heat flux density of 36,111 W/m^2^ was applied to the bottom of the microchannel manifold structure.

Based on the literature review [27,28], the Realizable k–ε turbulence model was adopted, and the simulation results of the chip junction temperature only objectively present the outcomes after applying this model. The accuracy of the model will be further verified through experiments in future work to improve the research conclusions. The steady-state simulation solved the energy equation with the Realizable *k*–*ε* model to capture coupled turbulent heat transfer. All fluid–solid interfaces were treated as no-slip, stationary walls. The entire domain was initialized at 26.5 °C. The specific equations used are as follows:

(2) $$\frac{\partial \left(\mathrm{pk}\right)}{\partial t}+\frac{\partial \left(\mathrm{pk}u_{i}\right)}{\partial x_{i}}=\frac{\partial }{\partial x_{j}}\left[\left(\mu +\frac{\mu_{t}}{\sigma_{k}}\right)\frac{\partial k}{\partial x_{j}}\right]+G_{k}-\rho \varepsilon$$

(3) $$\frac{\partial \left(p\varepsilon \right)}{\partial t}+\frac{\partial \left(p\varepsilon u_{i}\right)}{\partial x_{i}}=\frac{\partial }{\partial x_{j}}\left[\left(\mu +\frac{\mu_{t}}{\sigma_{\varepsilon }}\right)\frac{\partial \varepsilon }{\partial x_{j}}\right]+\rho C_{1}E\varepsilon -\rho C_{2}\frac{\varepsilon^{2}}{k+\sqrt{v\varepsilon }}$$

where *k* represents the turbulent kinetic energy, *ε* denotes the dissipation rate of turbulent kinetic energy, $\mu_{t}$ is the turbulent viscosity coefficient, while *ρ*, *μ*, *u*, and *ν* respectively represent the fluid density, kinematic viscosity coefficient, flow velocity, and dynamic viscosity coefficient. $G_{k}$ is the turbulence kinetic energy generation term caused by the mean velocity gradient, and $\sigma_{k}$ and $\sigma_{\varepsilon }$ are the Prandtl numbers corresponding to the turbulent kinetic energy *k* and the dissipation rate *ε*, respectively.

### 2.3. Intelligent Co-Optimization for Thermal Management

First, the orthogonal experimental method was employed to systematically design microchannel parameters to obtain diverse sample data. In this study, the L_25_ (5^6^) orthogonal array was selected, which accommodates 6 factors, each with 5 levels, resulting in a total of 25 experimental groups. Through orthogonal experiments, representative microchannel structure samples were efficiently generated, providing a rich data foundation for subsequent machine learning model training and optimization.

A Back-Propagation Neural Network (BPNN) model was constructed using the Nftool toolbox in MATLAB software (MATLAB R2024a), with microchannel parameters serving as input features to the input layer. The number of nodes in the intermediate hidden layer, which significantly affects the network’s learning capability and prediction accuracy, was determined using Kolmogorov’s theorem, expressed by the formula M = 2N + 1, where M and N represent the hidden layer and input layer nodes respectively. This configuration ensures optimal network complexity for capturing intricate input/output relationships while avoiding overfitting. Orthogonal experimental data formed the sample data and were split into 250 training sets and 25 test sets to validate generalization capability. All data were processed as input/output variables using the purelin transfer function and gradient descent training method. The optimization variables included six parameters: coolant type, channel width, channel depth, inlet/outlet position, and flow velocity (Table 4), with chip junction temperature minimization as the objective function. Network thresholds and inter-node weights were iteratively adjusted through error function minimization to enhance prediction accuracy.

Multi-parameter optimization was executed in MATLAB using the Statistics and Machine Learning Toolbox and Global Optimization Toolbox. The pre-trained random forest model and data standardization parameters were initially loaded, followed by defining an objective function that standardized input decision variables for random forest prediction. Six decision variables were configured with specified types (integer/continuous) and value ranges. Particle Swarm Optimization (PSO) parameters, including swarm size and maximum iterations, were set before executing PSO to identify constraint-compliant solutions that minimize the objective function. The optimal variable combination and corresponding predicted objective value were subsequently output. A grid search method evaluated each parameter set through cross-validation, ultimately determining the configuration achieving the minimal chip junction temperature for optimal microchannel design.

## 3. Results and Analysis

### 3.1. Collaborative Parameter Optimization of LVBF-MMS Cooling System

**Figure 3:**
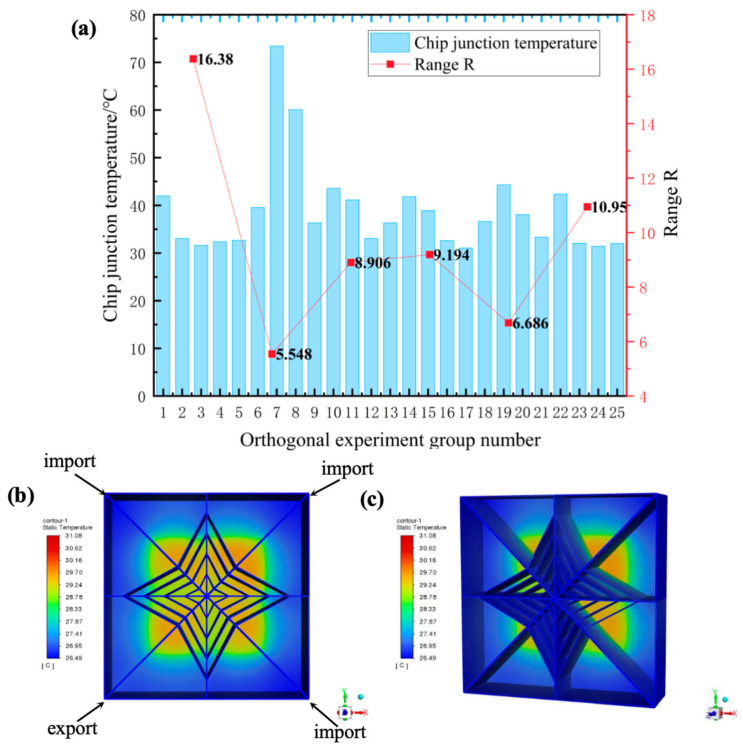
(**a**) Orthogonal experimental design and results of L_25_ (5^6^). (**b**) Schematic diagram of simulation result of optimal chip junction temperature by orthogonal experimental design. (**c**) Three-dimensional schematic diagram of simulation result of optimal chip junction temperature by orthogonal experimental design.

Increased microchannel depth-to-width ratios enhance convective cooling by elevating flow velocity, significantly reducing work temperatures [29,30,31]. Fractal flow-splitting structures (e.g., hydrofoil-shaped distributors) implemented at inlets optimize flow uniformity when combined with strategic inlet/outlet positioning [32,33,34,35]. These configurations suppress vortex generation while minimizing pressure losses [36,37], collectively improving thermal efficiency. Based on these thermal enhancement mechanisms, a six-factor five-level orthogonal array (L_25_ (5^6^)) was implemented to systematically identify optimal parameters, with the thermal resistance results concluded in Table 5 and Figure 3a. As demonstrated, the optimal parameter combination was determined as follows: working fluid of 50% ethylene glycol, flow velocity of 4 m/s, microchannel diameter of 0.75 mm, microchannel depth of 25 mm, inlet/outlet configuration III (inlets: left1, right1, right2; outlet: left2), and heat sink base thickness of 0.5 mm. This configuration achieved a minimum junction temperature of 31.08 °C (Group 17, Figure 3b,c). Notably, while covering merely 1/625th of possible combinations (15,625 total), this empirical optimum provides critical baseline data for subsequent theoretical optimization in thermal design refinement.

To verify grid independence, three sets of progressively refined polyhedral meshes were employed to ensure grid independence: coarse (370,441 cells), medium (492,938 cells), and fine (775,924 cells), all featuring wall inflation layers. The predicted chip junction temperatures converged to 37.36 °C, 37.60 °C, and 37.52 °C, respectively, with a maximum deviation of only 0.24 °C between the coarsest and finest grids, confirming mesh independence. For the optimal configurations identified in the orthogonal experiments, a refined mesh comprising 1,531,022 polyhedral cells was adopted, with three boundary layers (growth ratio of 1.2) and localized hexahedral refinement (minimum size of 0.1 mm) at fractal bifurcations of leaf veins and peripheral microchannel regions. This final mesh achieved a grid convergence index below 1.5% while maintaining computational efficiency.

Thermal resistance is defined as R=l/KS, where l is the length of heat conduction, S is the cross-sectional area of heat flow, and K is the thermal conductivity of the material. In the formula, l refers to the path length of heat flow along the main heat conduction direction (e.g., the thickness of the solid wall between the heat source and the microchannel), and S refers to the cross-sectional area perpendicular to the heat flow direction (e.g., the contact area between the heat source and the heat sink). In design, efforts should be made to reduce l (thin structure), increase S (large contact area), and select high-K materials. Meanwhile, optimizing the channel structure to reduce convective thermal resistance is necessary to lower the total thermal resistance and improve heat dissipation efficiency.

The chip to be cooled maintains reliable contact with the copper layer cover plate on the microchannel heat sink. The heat generated by the chip undergoes heat exchange with the coolant in the microchannel heat sink, and the heat is removed by the flowing coolant to achieve chip cooling. During heat exchange between the heat source and the embedded microchannel heat sink, a “resistance” effect occurs, termed thermal resistance, which can be analogous to electrical resistance in electronics. This process exhibits thermal resistance (analogous to electrical resistance), where lower resistance enables higher cooling efficiency. Thermal resistance is expressed as follows:

(4) $$R_{th}=\frac{T_{max}-T_{in}}{q}$$

where $T_{max}$ is the maximum temperature of the heat source surface, $T_{in}$ is the inlet water temperature, and *q* is the total heat source. Thermal resistance was obtained from the simulation, where the maximum chip junction temperature was 31.08 °C, representing the maximum temperature $T_{max}$ at the heat source end. The fluid inlet temperature was set to 26.5 °C, representing the minimum temperature $T_{in}$ at the heat dissipation end. The radiator microchannel had a diameter of 0.75 mm, with a flow area A of π$\;\times {\left(\frac{0.75}{2}\right)}^{2}\approx$0.44 mm^2^ and an inlet velocity *V* of 4 m/s.

(5) q=A×V=0.44×4000= 1.76 mL/min

(6) $$R_{th}=\frac{T_{max}-T_{in}}{q}=\frac{31.08-26.5}{1.76}\;=\;2.6^{\;\circ }\mathrm{C/W}$$

**Figure 4:**
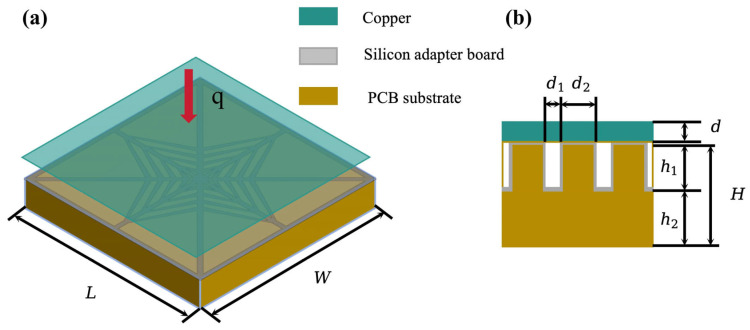
The principle and structure of the embedded microchannel heat sink: (**a**) 3D view (length L and width W of the microchannel heat sink); (**b**) cross-sectional view (height H; Cu layer thickness d; groove width $d_{1}$; rib width $d_{2}$; base thickness $h_{1}$; groove height $h_{2}$).

To further construct a thermal resistance network model for embedded microchannels, the three-dimensional heterogeneously integrated AI chip architecture was conceptualized as a heterogeneous stack of functionally diverse 2D chips, with heat generated primarily within the active layers of each tier. Thermal management optimization critically depends on: (1) embedding microchannels within silicon substrates, and (2) establishing precise thermal resistance networks for these regions. For the single micro-element of the secondary flow channel shown in Figure 4, the embedded body position defines a boundary that yields two distinct heat conduction paths in single-heat-source systems: the first conducts heat to the substrate for dissipation, while the second transfers heat to the coolant via convective heat transfer at fluid–wall interfaces and fluid flow. Based on this dual-path model, each secondary flow channel is divided transversely into three sections, with the convective resistance between fluid and walls treated as an integrated component. The heat transfer follows a series network consisting of microchannel-embedded body conduction resistance → fluid–wall convective resistance → fluid thermal resistance, with their sum constituting the total thermal resistance of the network model.

**Figure 5:**
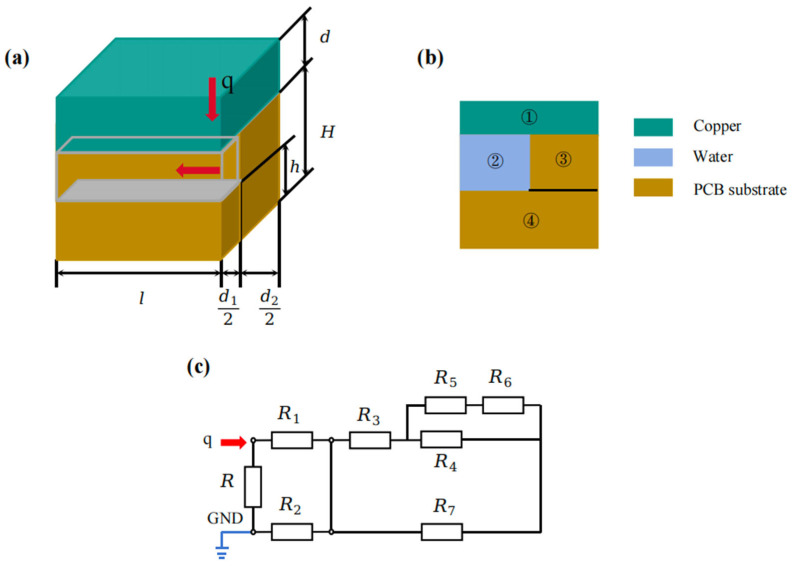
Cross-sectional schematic diagram and thermal resistance model of half of the microchannel region without a nickel plating layer. (**a**) Schematic diagram of the structure and heat transfer principle of the half-microchannel region in the embedded microchannel heat sink. (**b**) Cross-sectional schematic diagram (① copper layer, ② cooling liquid, ③ rib of the microchannel heat sink, ④ base of the microchannel heat sink). (**c**) Thermal resistance model. (The red arrow indicates the direction of heat flow).

**Figure 6:**
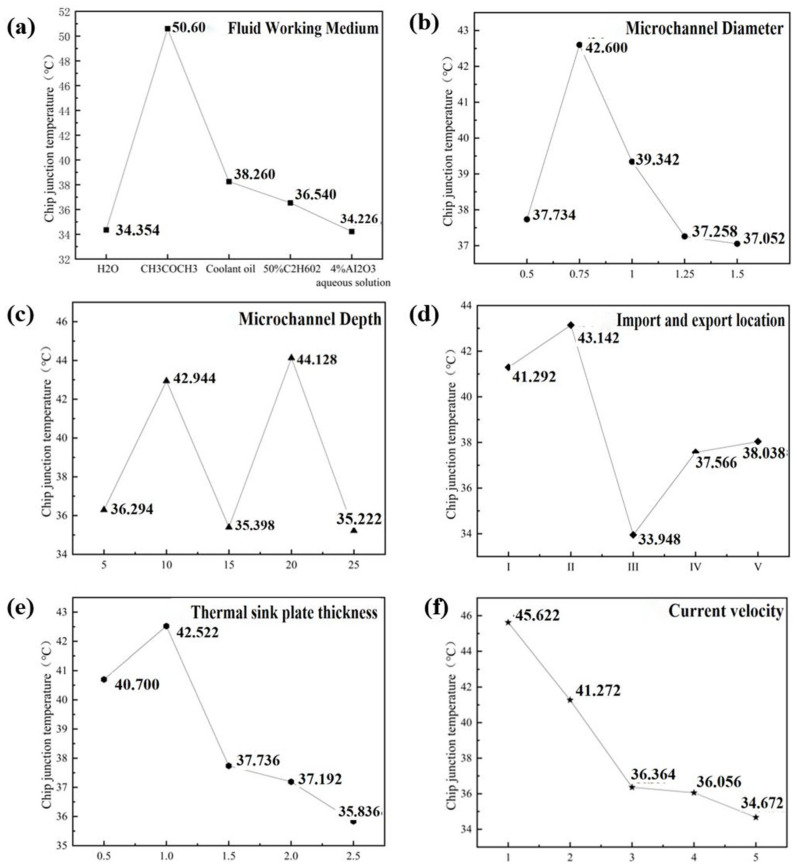
The trend of influence of each factor on chip junction temperature: (**a**) fluid working medium; (**b**) microchannel diameter; (**c**) microchannel depth; (**d**) import and export location; (**e**) thermal sink plate thickness; (**f**) current velocity.

Based on the heat transfer modes in the microchannel heat sink, its thermal resistance can be classified into conductive thermal resistance, convective heat transfer resistance, and fluid flow resistance. Considering structural symmetry, for thermal analysis of the embedded microchannel heat sink structure (Figure 4), half of a channel and rib were selected for this study, as illustrated in Figure 5a. Its cross-sectional schematic is shown in Figure 6a. The main heat transfer paths include: heat conduction through the copper cover layer (①), heat conduction through the rib of the microchannel heat sink (③), convective heat transfer between the copper cover layer (①) and the coolant (②), heat conduction through the base of the microchannel heat sink (④), and convective heat transfer between the coolant (②) and the base of the microchannel heat sink (④). The corresponding thermal resistance model is presented in Figure 6b, which can be represented as a series-parallel combination of various thermal resistances. Hence, according to Figure 6b, the total thermal resistance of half of the computational domain can be obtained as

(7a) $$R_{th}=R∥\left\{R_{1}+R_{2}+\left[\left(\left(R_{5}+R_{6}\right)∥R_{4}\right)+R_{3}\right]∥R_{7}\right\}$$

When the bypass thermal resistance $\;R\gg R_{1}+R_{2}+\left[\left(\left(R_{5}+R_{6}\right)∥R_{4}\right)+R_{3}\right]∥R_{7}$, the thermal resistance $R_{th}$ in Equation (7) is expressed as

(7b) $$R_{th}=R_{1}+R_{2}+\left[\left(\left(R_{5}+R_{6}\right)∥R_{4}\right)+R_{3}\right]∥R_{7}$$

When the bypass thermal resistance $\;R\ll R_{1}+R_{2}+\left[\left(\left(R_{5}+R_{6}\right)∥R_{4}\right)+R_{3}\right]∥R_{7}$, the thermal resistance $R_{th}$ in Equation (7) is expressed as

(7c) $$R_{th}=R$$

where *R*_1_ is the conduction thermal resistance of the copper cover plate, which is very small due to copper’s high thermal conductivity. It is proportional to thickness and inversely proportional to thermal conductivity (the dominant factor), rib width, groove width, and flow length. *R*_2_, the fluid flow thermal resistance, is inversely proportional to the coolant’s specific heat capacity, density, flow velocity (the dominant factor), and channel cross-sectional area. Higher flow velocity substantially reduces *R*_2_. *R*_3_, the rib wall conduction thermal resistance, is proportional to groove height and inversely proportional to rib wall thermal conductivity (the dominant factor), rib width, and channel length l. Due to silicon’s high rib wall thermal conductivity, *R*_3_ is small. *R*_4_, the convective heat transfer thermal resistance between the rib wall and the fluid, is inversely proportional to the convective heat transfer coefficient (the dominant factor), groove height, and channel length. The convective heat transfer coefficient is highly sensitive to flow regime, fluid properties, and surface geometry, making *R*_4_ the most critical resistance component. R_5_, the convective heat transfer resistance between the substrate and the fluid, is inversely proportional to the convective heat transfer coefficient (the dominant factor), groove width, and channel length. R_6_, the substrate conduction thermal resistance, is proportional to base thickness and inversely proportional to base material thermal conductivity (the dominant factor), rib width, groove width, and channel length. Due to TU-752’s low thermal conductivity, R_6_ is relatively large. R_7_, the convective heat transfer resistance between the cover plate and fluid, is inversely proportional to the convective heat transfer coefficient (the dominant factor), total width, and channel length.

The pressure drops at the inlet and outlet are

(8) $${∆P}_{in}=K_{in}\times \frac{\rho u_{in}}{2}=0.12\times \frac{1073.4\times 4}{2}=257.62\;Pa$$

(9) $${∆P}_{out}=K_{out}\times \frac{\rho u_{out}}{2}=\frac{1073.4\times 4}{2}=2146.8\;Pa$$

where ρ is the mass density of the coolant, $K_{in}$ is the loss coefficient for the sudden contraction inlet, and $K_{out}$ is the loss coefficient for the sudden expansion outlet. Typically, for a right-angle inlet, $K_{in}$ = 0.5. For a rounded inlet, $K_{in}$ = 0.03. For a micro-rounded inlet, $K_{in}$ = 0.12. $K_{out}$ is the easiest to determine and approximately equal to 1.

The frictional pressure drop in the microchannel is

(10) $${∆P}_{c}=f\frac{\rho u}{2}\left(\frac{L}{D_{h}}\right)$$

where

(11) $$f=\frac{K\left(\infty \right)}{4\left[L/\left(D_{h}\times Re\right)\right]}+\frac{96/Re}{{\left[1+\frac{1}{\alpha }\right]}^{2}\left[1-\frac{192}{\pi^{5}\alpha }\sum_{n=1,3,5}^{\infty }\left(\frac{tanh\left(n\pi \alpha /2\right)}{n^{5}}\right)\right]}$$

(12) $$K\left(\infty \right)=0.6796+1.2197\alpha +3.3089\alpha^{2}-9.5921\alpha^{3}+8.9089\alpha^{4}-2.9959\alpha^{5}$$

(13) $$D_{h}=\frac{4S}{C}=\frac{4\times 27}{21.9017}\approx 4.93\;mm=0.00493\;m$$

(14) $$Re=\frac{\rho uD_{h}}{\mu }=\frac{1073.4\times 4\times 0.00493}{0.00394}\approx 5372.45$$

where f is the friction coefficient, *ρ* is the fluid density, L is the flow path length, u is the velocity magnitude, and $D_{h}$ is the hydraulic diameter. $K\left(\infty \right)$ is the constant Hagenbach factor, Re is the Reynolds number, and μ is the viscosity of the coolant. The constant Hagenbach factor is a function of the aspect ratio α (0 < α < 1). S is the channel area, and C is the channel perimeter. It can be seen from the equation that the frictional pressure drop is proportional to the friction coefficient f, flow length L, and velocity magnitude u, and inversely proportional to the hydraulic diameter $D_{h}$.

Aspect ratio of the microchannel:

$$\alpha =\frac{0.75}{25}=0.03,\;\mathrm{obtain}\;K\left(\infty \right)\approx 0.72,\;f=217.68,\;{∆P}_{c}=2076.07\mathrm{Pa}$$

Total pressure drop:

(15) $$∆P={∆P}_{in}+{∆P}_{out}+{∆P}_{c}=257.62+2146.8+2076.07=4480.49\;Pa$$

As the inlet velocity of the fluid working medium increases, the pressure drop of the fluid in microchannels with different diameters increases; when the inlet velocity of the fluid working medium is the same, the pressure drop between the inlet and outlet of the fluid in the microchannel increases as its diameter decreases. When the fluid velocity increases, the liquid loss in the channel is proportional to the square of the liquid velocity, thus increasing the pressure drop. When the channel diameter decreases, on the one hand, the number of microchannels that can be laid in the same area increases; on the other hand, the hydraulic diameter of the microchannel decreases, which increases the friction factor between the fluid and the wall, resulting in an increase in pressure drop.

### 3.2. Parametric Sensitivity Analysis via Range and Variance Methods

The synergistic use of range and variance analyses identifies optimal parameters by evaluating factor influence through averaged level responses and range magnitudes, with comprehensive results revealing key thermal performance insights. Fundamentally, optimization is a mathematical process of finding an extremum (minimum or maximum) for an objective function within a design space defined by variables and constraints. These variables shape the objective function, guide the optimization strategy, and inform constraint settings, which collectively confine solutions to a feasible region. As the core of the process, the objective function’s extremum is sought via search algorithms that navigate the constrained design space to locate optimal variable values.

Considering the impact of structural dimensions and microchannel design on the chip junction temperature, this section presents an orthogonal experiment to investigate the effects of multiple factors (coolant fluid, microchannel diameter, microchannel depth, inlet/outlet position, heat spreader thickness, flow velocity) at multiple levels on the junction temperature of a high-performance AI chip based on chiplet heterogeneous integration and embedded with a bio-inspired fractal microchannel manifold structure. The range method will be used to identify the optimal level combination, with chip junction temperature as the indicator. The constraints were as follows: coolant fluid: selected from H_2_O, acetone, cooling oil, 50% ethylene glycol, or 4% Al_2_O_3_ aqueous solution; microchannel diameter: selected from a range of [0.5, 1.5] mm; microchannel depth: selected from a range of [5, 25] μm; inlet/outlet position: selected from different positions with [1, 3] inlets/outlets; heat spreader thickness: selected from a range of [0.5, 2.5] mm; flow velocity: selected from a range of [1, 5] m/s. The extremum of the objective function is achieved when the design variables satisfy the following criterion:

(16) $$R_{i}=max\left[\bar{K_{i1}},\bar{K_{i2}},\bar{K_{i3}},\cdots \bar{K_{in}}\right]-min\left[\bar{K_{i1}},\bar{K_{i2}},\bar{K_{i3}},\cdots \bar{K_{in}}\right]$$

where $\bar{K_{ij}}$ represents the average value of the test indicator under the *j*-th test level of the *i*-th experimental factor. $R_{i}$ is the difference between the maximum and minimum values of $\bar{K_{ij}}$ across all levels of the *i*-th factor, indicating the magnitude of influence of the *i*-th factor on the test indicator. The results are presented in Table 6 and Figure 6. As shown in Figure 6a, working fluids such as H_2_O, coolant oil, 50% ethylene glycol aqueous solution, and 4% Al_2_O_3_ nanofluid achieved chip junction temperatures (*T_j_*) below 40 °C, while acetone’s low specific heat capacity (1.47 kJ/kg·K) induced abrupt temperature surges due to inadequate heat dissipation.

Figure 6b depicts the effect of microchannel diameter (0.5–1.5 mm) on the maximum *T_j_*. With increasing diameter, the temperature first rises and then declines. Specifically, the temperature increases from 37.734 °C to 42.6 °C within 0.5–0.75 mm but decreases from 42.6 °C to 37.052 °C within 0.75–1.5 mm. This nonlinear trend indicates that while larger diameters enhance contact area with the heat source and reduce *T_j_*, their impact on chip cooling is not significant. Therefore, a microchannel diameter of 1.25 mm is recommended, as further increases beyond this value yield negligible temperature reductions. Figure 6c shows the trend of microchannel depth (5–25 mm) on the maximum *T_j_*. The temperature exhibits a complex pattern: peaking at 10 mm and 20 mm depths, while reaching troughs at 5 mm, 15 mm, and 25 mm. The lowest temperature (35.222 °C) occurs at 25 mm. This may be attributed to improved heat dispersion beyond 20 mm depth, facilitating stable temperature reduction. While depths of 5 mm, 15 mm, and 25 mm demonstrate effective cooling, deeper channels do not universally guarantee superior performance due to increased flow resistance and pumping power penalties. Crucially, thermal optimization requires balancing depth with aspect ratio (depth/width), where high-aspect-ratio microchannels (>30:1) maximize convective heat transfer by augmenting the fluid–solid contact area while minimizing axial thermal resistance. For inlet/outlet configurations (Figure 6d), Configuration III (3 inlets/1 outlet) achieves optimal cooling by balancing inflow volume, while asymmetric designs (e.g., fewer inlets) restrict coolant supply, elevating temperatures. Base thickness variations (Figure 6e) reveal a *T_j_* peak at 42.522 °C (1 mm thickness), followed by decline to 35.836 °C (2.5 mm) as thicker bases overcome initial convective penalties through enhanced lateral conduction, recommending ≥1.5 mm for sustained benefits. Figure 6f displays the influence of microchannel flow velocity (1–5 m/s) on the maximum *T_j_*. The temperature steadily decreases from 45.622 °C to 34.672 °C with increasing velocity, as faster flow absorbs heat more rapidly. Although higher velocities improve cooling, temperature reduction plateaus beyond 3 m/s.

In minimization problems, it is necessary to continuously search for the global minimum, making it highly susceptible to becoming trapped in local minima, particularly when these local minima are prominent. The criterion for determining the significance of a factor is as follows: when the F-value < $F_{0.05}$, the factor is considered non-significant and denoted by *; when $F_{0.05}$ < F-value < $F_{0.01}$, the factor is considered sub-significant; when F-value $>F_{0.01}$, the factor is considered relatively significant and denoted by **; when F-value $\gg F_{0.01}$, the factor is considered significant and denoted by ***. Moreover, a higher F-value indicates a greater degree of importance for the factor.

Analysis of variance (ANOVA) quantifies factor significance through *F*-statistic construction, computed via sum of squares, degrees of freedom, and variance decomposition (Table 7). Significance hierarchy is determined by *F*-value thresholds relative to critical values: when *F*-value < F_0.05_(4,20), the factor is non-significant, denoted by *; when F_0.05_(4,20) < F-value < F_0.01_(4,20), it is a marginally significant factor, denoted by **; when F-value > F_0.01_(4,20), it is a significant factor, denoted by ***; and when F-value is significantly greater than F_0.01_(4,20), it is a highly significant factor, denoted by ****. A larger F-value correlates with greater factor influence. As shown in Table 8, among the five factors in the ANOVA, factor A (fluid working medium) is a highly significant factor, factors B (microchannel depth) and E (heat sink base thickness) are marginally significant factors, while factors D (inlet/outlet position) and F (flow velocity) are insignificant factors. This result aligns with the range analysis conclusion, demonstrating methodological consistency. The synergistic application of range analysis and ANOVA thus robustly identifies dominant thermal drivers (fluid selection > depth > base thickness) while establishing Configuration III, with 50% ethylene glycol, 25 mm depth, and 0.5 mm base, as the empirically optimal combination for minimizing junction temperature.

### 3.3. Parameter Optimization Method Based on Machine Learning

The intelligent optimization employing BPNN and the PSO algorithm identifies the optimal chip junction temperature parameters from $5^{6}$ different combinations, providing an intelligent automation framework that solves complex collaborative optimization problems beyond the reach of manual trial-and-error. Significant differences exist in the optimal solution indicators corresponding to different optimization methods, and the optimal values of design variables exhibit similar variation trends. While directly evaluating the best parameters via CFD requires setting different objective functions, the neural network optimization method offers universality and is applicable to future in-depth research on novel microchannel structures, such as porous-embedded channels.

**Figure 7:**
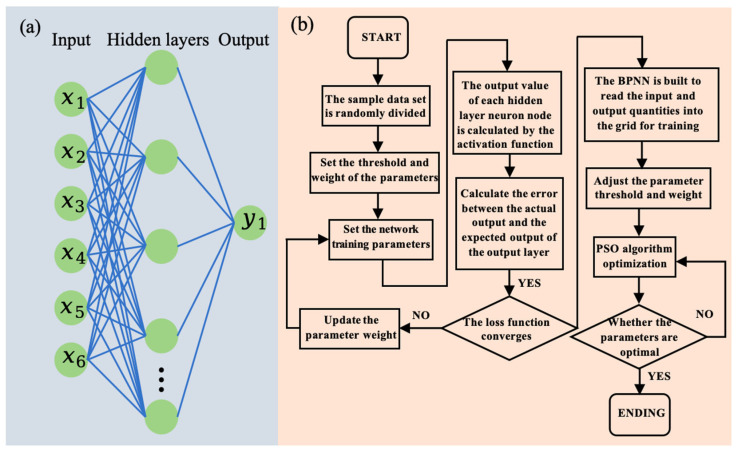
(**a**) Structure of neural network model ($x_{1}$: fluid working medium; $x_{2}$: microchannel diameter; $x_{3}$: microchannel depth; $x_{4}$: import and export location; $x_{5}$: thermal sink plate thickness; $x_{6}$: current velocity; $y_{1}$: junction temperature). (**b**) Machine learning multi-objective optimization flowchart.

Based on orthogonal experiment results, the prediction accuracy and optimization effectiveness are enhanced by integrating BPNN with the PSO algorithm. The specific flowchart is illustrated in Figure 7. First, the sample data is randomly divided into training and testing sets at a 10:3 ratio. After initializing network parameter thresholds and weights, parameters are updated through back-propagation based on the error between the actual and expected outputs of the output layer, with prediction results determined by the convergence of the loss function. Then, a BPNN is constructed, where the working fluid, microchannel diameter, microchannel depth, inlet/outlet positions, thermal sink plate thickness, and flow velocity serve as input variables, while the chip junction temperature acts as the output variable for network training. Finally, the PSO algorithm iteratively optimizes parameters; if suboptimal, particle positions continue updating until the optimal parameter model is achieved. Concurrently, decision variable ranges and constraints are defined: the working fluid (Variable A) is selected from pure water, acetone, cooling oil, 50% 50%C_2_H_6_O_2_, or 4% Al_2_O_3_ aqueous solution; the microchannel diameter (Variable B) is within [0.25, 1.75] mm; the microchannel depth (Variable C) is within [1, 30] mm; the inlet/outlet positions (Variable D) are chosen from four predefined coordinate sets; thermal sink plate thickness (Variable E) is within [0.25, 2.75] mm; flow velocity (Variable F) is within [0.5, 5.5] m/s. The grid search method combines and optimizes these parameters, ultimately yielding the optimal microchannel design that minimizes chip junction temperature.

**Figure 8:**
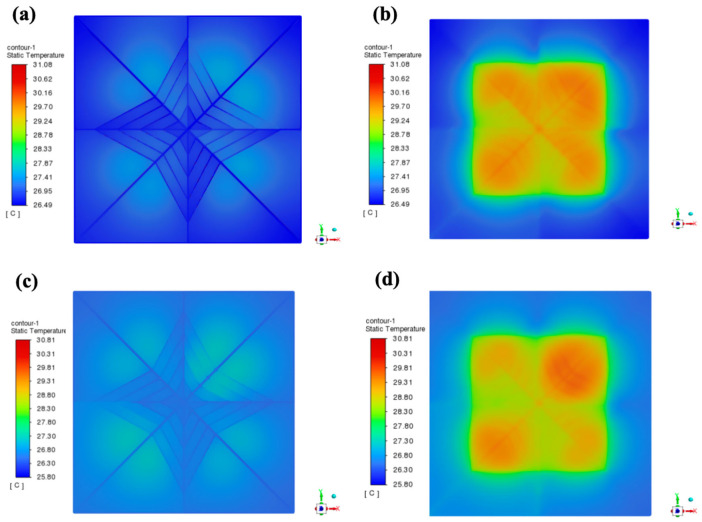
Slice simulation diagram of optimal result from orthogonal test. (**a**) Microchannel interior. (**b**) Microchannel near heat source (neural network machine learning training sample input for optimal result). (**c**) Microchannel interior. (**d**) Microchannel near heat source.

**Figure 9:**
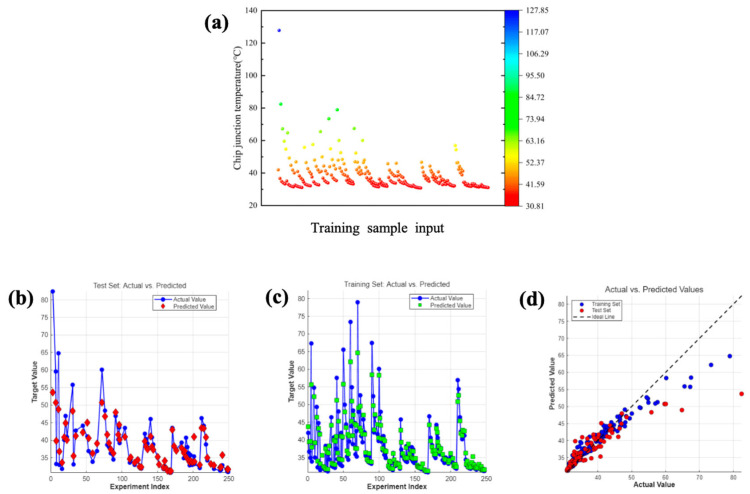
(**a**) Scatter plot of neural network training input values. (**b**) Comparison between test samples and neural network training output values. (**c**) Comparison between training samples and neural network training output values. (**d**) Comparison of scatter plots between test samples and training samples.

To bring the optimization results closer to the actual values, among the 5^6^ different parameter combinations derived from a six-factor five-level orthogonal experiment, the 250 sets of training sample data were used to select the two most significant factors with the highest critical Fα values identified through orthogonal experimental analysis of variance—namely the working fluid and flow velocity—as the primary variables. By varying the materials of the working fluid and the flow velocity, parameter combinations were generated with the remaining four factors and simulated. The resulting data served as training samples for subsequent optimization. The optimal orthogonal experiment results are displayed in the cross-section simulation views shown in Figure 8a,b, with a chip junction temperature of 31.08 °C. The simulation results for the optimal solution using 250 sets of training samples from the neural network machine learning are presented in Figure 8c,d, yielding a chip junction temperature of 30.81 °C. A scatter plot of the 250 training samples is shown in Figure 9a, with simulation results of chip junction temperatures for different combinations predominantly concentrated between 30–50 °C. Prediction scatter plots for the test samples versus training samples in Figure 9b–d fully demonstrate the high fitting degree of the simulation results. Subsequently, machine learning and PSO yielded significantly refined prediction accuracy (validated in Figure 8), culminating in an optimal thermal solution: 50% C_2_H_6_O_2_, fluid at 4.43 m/s flow velocity, 0.72 mm microchannel diameter, 29.91 mm depth, Configuration III inlets/outlet (inlets: left1, right1, right2; outlet: left2), and 0.43 mm baseplate thickness. These variables are numerical optimization targets rather than verified directly manufacturable designs. For the physical feasibility of the optimized design parameters, experimental verification will be conducted in the future. Ultimately, SolidWorks reconstruction and Ansys Fluent simulation confirmed a 30.97 °C chip junction temperature, verifying the reliability of this integrated methodology.

**Figure 10:**
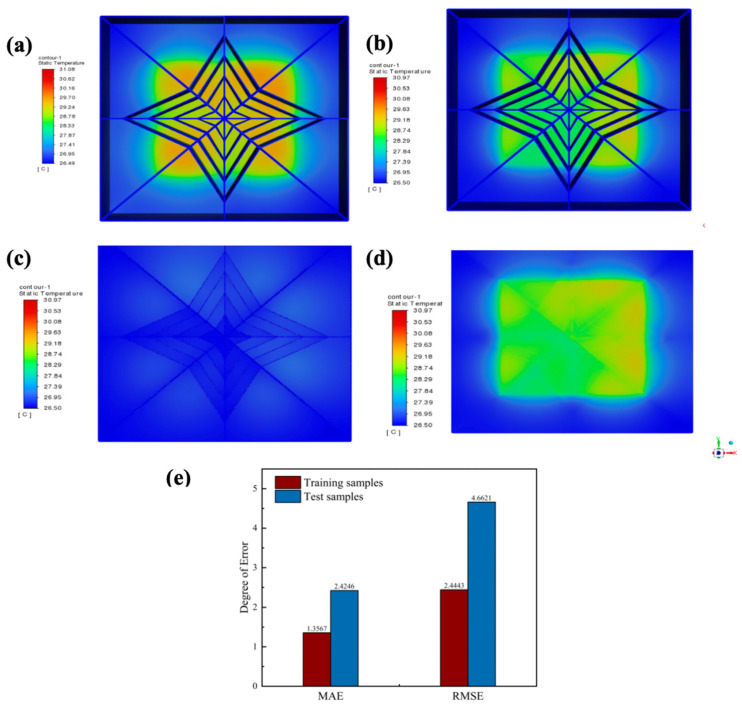
(**a**) Simulation diagram of optimal parameters for test samples. (**b**) Simulation diagram of optimal parameters for training samples. (**c**) Simulation of the microchannel cross-section inside the training sample. (**d**) Simulation of the microchannel cross-section near the heat source in the training sample. (**e**) Error index of multi-objective parameter optimization method based on machine learning.

In model evaluation, the discrepancy between training set and test set performance serves as a crucial indicator for assessing model generalization capability. High training metrics with low test metrics suggest overfitting, while the inverse indicates underfitting. Therefore, the optimal model configuration minimizes this performance discrepancy while maintaining high accuracy on both datasets. A test value as high as 0.9137 demonstrates that the model successfully captures the vast majority of underlying patterns and relationships within the test data. The chip junction temperature with optimal parameters was 31.08 °C for the test sample, as shown in Figure 9a; the chip junction temperature with optimal parameters was 30.97 °C for the training sample, as shown in Figure 10b–d. The results demonstrate higher accuracy when the significant factors identified through orthogonal experiments are used as the main variables. To quantify prediction accuracy, this study employed multiple error metrics for evaluation. Both the Mean Absolute Error (MAE) and Root Mean Square Error (RMSE) were utilized to measure prediction precision [38]. Prediction accuracy was quantified using multiple error metrics:

(17) $$\mathrm{RMSE}\;=\sqrt{\frac{1}{n}\sum_{i}^{n}{\left(y_{i}-\;{\hat{y}}_{i}\right)}^{2}}$$

(18) $$\mathrm{MAE}=\frac{1}{n}\sum_{i=1}^{n}\left|y_{i}-\;{\hat{y}}_{i}\right|$$

where, *n* represents the number of samples, ${\hat{y}}_{i}$ denotes the predicted value, and $y_{i}$ represents the actual value. The RMSE for the training samples was merely 2.4443 kcal/mol, with an MAE of 1.3567. For the test samples, the RMSE was 4.6621 kcal/mol, with an MAE of 2.4246. In complex systems like heat transfer, which are influenced by multiple factors, a mean absolute error of 2.4246 is considered highly favorable. As illustrated in Figure 10e, a comparison of the MAE and RMSE metrics corresponding to the test data clearly reveals the model’s superior performance under non-uniform thermal distribution conditions. This achievement facilitates the intelligent design of adaptive heat sources within LVBF-MMS.

## 4. Conclusions

This paper presents an embedded leaf-vein-inspired fractal microchannel thermal model tailored for heterogeneously integrated high-performance AI chips subjected to highly non-uniform heat loads. Anchored in a one-dimensional thermal-resistance network that couples fluidic and solid domains, the model unites a reconfigurable chiplet heat source with a full 3D chip thermal field, thereby eliminating the vertical cooling imbalance and hotspot formation inherent in conventional fixed heat sink schemes. Dense hierarchically bifurcated microchannels multiply the coolant-to-surface contact area and are optimized—via orthogonal design of experiments (range/ANOVA) cross-validated with machine-learning-driven PSO search—for diameter, depth, inlet/outlet placement, substrate thickness and flow rate. Under single-phase liquid cooling, the optimized topology delivers exceptional temperature uniformity, reducing peak junction temperature from 127.80 °C to 30.97 °C (a 76% drop), even under extreme heat-flux gradients, and provides a robust theoretical framework and design methodology for hotspot mitigation in heterogeneously integrated AI chips.

## Figures and Tables

**Table 1:** Microchannel manifold structural model parameters.

Category	Size	Material
Adapter board containing TSV	120 mm × 120 mm × 6 mm	Si
Bump	60 mm × 60 mm × 3 mm	60Sn40Pb
Solder ball	120 mm × 120 mm × 6 mm	60Sn40Pb
PCB substrate	120 mm × 120 mm × 10 mm	TU-752
ASIC	23 mm × 23 mm × 3 mm	Si
Power Chip	23 mm × 23 mm × 3 mm	Si
NPU	11 mm × 23 mm × 3 mm	Si
Sensor	11 mm × 23 mm × 3 mm	Si
HBM	11 mm × 23 mm × 3 mm	Si
I/O Chip	11 mm × 23 mm × 3 mm	Si

Note: Si: TSMC (Hsinchu Science Park, Taiwan, China); 60Sn40Pb: Mitsubishi Materials Corporation (Tokyo, Japan); TU-752: Mitsubishi Gas Chemical Company (Tokyo, Japan).

**Table 2:** Orthogonal experimental factors and levels of L_25_ (5^6^).

Factor		Fluid Working Medium (A)	Microchannel Diameter/mm (B)	Microchannel Depth/mm (C)	Import and Export Location (D)	Thermal Sink Plate Thickness/mm (E)	Current Velocity/m/s (F)
**Level**	1	H_2_O	0.5	5	I	0.5	1
	2	CH_3_COCH_3_	0.75	10	II	1	2
	3	Coolant oil	1	15	III	1.5	3
	4	50%C_2_H_6_O_2_	1.25	20	IV	2	4
	5	4%AI_2_O_3_aqueous solution	1.5	25	V	2.5	5

**Table 3:** Simulation parameters of the microchannel manifold structural model.

Category	Density /(kg·m^−3^)	Specific Heat Capacity /(J·kg^−1^·K^−1^)	Thermal Conductivity /(W·m^−1^·K^−1^)	Viscosity /(kg·m^−1^·s^−1^)
H_2_O	998.2	4182	0.6	0.001003
CH_3_COCH_3_	744	517	0.168	0.000226
Coolant oil	860	1900	0.15	0.0018
50%C_2_H_6_O_2_	1073.4	3281	0.38	0.00394
4%AI_2_O_3_ aqueous solution	1040	3900	0.75	1.3

**Table 4:** Inlet and outlet positions of microchannels.

I	II	III	IV	V
Inlet: Left1Right1	Inlet: Left1Right2	Inlet: Left1Right1Right2	Inlet: Left1	Inlet: Left1
Outlet: Left2Right2	Outlet: Left2Right1	Outlet: Left2	Outlet: Left2Right2Right1	Outlet: Right1

**Table 5:** L_25_ (5^6^) orthogonal experimental design and results.

Number of Columns	C1	C2	C3	C4	C5	C6	C7
Number	A	B	C	D	E	F	Chip Junction Temperature/°C
1	H_2_O	0.5	5	I	0.5	1	42.01
2	H_2_O	0.75	10	II	1	2	33.04
3	H_2_O	1	15	III	1.5	3	31.63
4	H_2_O	1.25	20	IV	2	4	32.40
5	H_2_O	1.5	25	V	2.5	5	32.69
6	CH_3_COCH_3_	0.5	10	III	2	5	39.55
7	CH_3_COCH_3_	0.75	15	IV	2.5	1	73.45
8	CH_3_COCH_3_	1	20	V	0.5	2	60.10
9	CH_3_COCH_3_	1.25	25	I	1	3	36.32
10	CH_3_COCH_3_	1.5	5	II	1.5	4	43.61
11	Coolant oil	0.5	15	V	1	4	41.17
12	Coolant oil	0.75	20	I	1.5	5	33.07
13	Coolant oil	1	25	II	2	1	36.32
14	Coolant oil	1.25	5	III	2.5	2	41.84
15	Coolant oil	1.5	10	IV	0.5	3	38.90
16	50%C_2_H_6_O_2_	0.5	20	II	2.5	3	32.61
17	50%C_2_H_6_O_2_	0.75	25	III	0.5	4	31.08
18	50%C_2_H_6_O_2_	1	5	IV	1	5	36.64
19	50%C_2_H_6_O_2_	1.25	10	V	1.5	1	44.32
20	50%C_2_H_6_O_2_	1.5	15	I	2	2	38.05
21	4%AI_2_O_3_aqueous solution	0.5	25	IV	1.5	2	33.33
22	4%AI_2_O_3_aqueous solution	0.75	5	V	2	3	42.36
23	4%AI_2_O_3_aqueous solution	1	10	I	2.5	4	32.02
24	4%AI_2_O_3_aqueous solution	1.25	15	II	0.5	5	31.41
25	4%AI_2_O_3_aqueous solution	1.5	20	III	1	1	32.01

**Table 6:** Analysis of junction temperature range for chips.

Factor		A	B	C	D	E	F
**Calculation item**	$K_{1}$	171.77	188.67	181.47	206.46	203.5	228.11
	$K_{2}$	253.03	213	214.72	215.71	212.61	206.36
	$K_{3}$	191.3	196.71	176.99	169.74	188.68	181.82
	$K_{4}$	182.7	186.29	220.64	187.83	185.96	180.28
	$K_{5}$	171.13	185.26	176.11	190.19	179.18	173.36
	$\bar{K_{1}}$	34.354	37.734	36.294	41.292	40.7	45.622
	$\bar{K_{2}}$	50.606	42.6	42.944	43.142	42.522	41.272
	$\bar{K_{3}}$	38.26	39.342	35.398	33.948	37.736	36.364
	$\bar{K_{4}}$	36.54	37.258	44.128	37.566	37.192	36.056
	$\bar{K_{5}}$	34.226	37.052	35.222	38.038	35.836	34.672
Range R		16.38	5.548	8.906	9.194	6.686	10.95
Factors are primary and secondary		A > F > D > C > E > B					
Good combinations		A4	B2	C5	D3	E1	F4

**Table 7:** Chip junction temperature results and variance analysis.

Number of Columns	C1	C2	C3	C4	C5	C6
Calculation item	A	Error column	C	D	E	F
$K_{1}$	171.77	188.67	181.47	206.46	203.5	228.11
$K_{2}$	253.03	213	214.72	215.71	212.61	206.36
$K_{3}$	191.3	196.71	176.99	169.74	188.68	181.82
$K_{4}$	182.7	186.29	220.64	187.83	185.96	180.28
$K_{5}$	171.13	185.26	176.11	190.19	179.18	173.36
$K_{1}^{2}$	29,504.9329	35,596.3689	32,931.3609	42,625.7316	41,412.25	52,034.1721
$K_{2}^{2}$	64,024.1809	45,369	46,104.6784	46,530.8041	45,203.0121	42,584.4496
$K_{3}^{2}$	36,595.69	38,694.8241	31,325.4601	28,811.6676	35,600.1424	33,058.5124
$K_{4}^{2}$	33,379.29	34,703.9641	48,682.0096	35,280.1089	34,581.1216	32,500.8784
$K_{5}^{2}$	29,285.4769	34,321.2676	31,014.7321	36,172.2361	32,105.4724	30,053.6896
T = 969.93						

**Table 8:** Analysis of variance table for orthogonal test results of chip junction temperature.

Source of Variance	Sum of Squared Deviations	Degrees of Freedom	Root Mean Square	F-Number	Critical Value Fα	Significance
Fluid working medium	927.345944	4	231.836486	8.706104873	F_0.05_(4,20) = 2.87	***
Microchannel depth (mm)	381.080024	4	95.270006	3.577653707	F_0.01_(4,20) = 4.43	**
Import and export location	253.541464	4	63.385366	2.380296792		*
Thermal sink plate thickness (mm)	149.831504	4	37.457876	1.406647428		*
Current velocity (m/s)	415.772224	4	103.943056	3.903350857		**
Microchannel diameter (mm) (Error e)	106.516744	4	26.629186			
Summation	2234.087904	24				

## Data Availability

The original contributions presented in this study are included in the article/Supplementary Material. Further inquiries can be directed to the corresponding author.

## Supplementary Materials

The following supporting information can be downloaded at: https://www.mdpi.com/article/10.3390/ma19040679/s1, Figure S1: Workflow diagram for multi-parameter optimization of bionic fractal microchannel structure, Figure S2: Planar layouts of four microchannel structures, Figure S3: Meshing of embedded venation biomimetic fractal microchannel manifold structures, Figure S4: Schematic diagram of primary and auxiliary channels in microchannel-embedded body (the region delineated by blue dashed lines represents the main flow channel, while the light blue shaded area indicates the secondary flow channel), Figure S5: Grid independence verification, Table S1: Dimensions of 3D stacked structural model, Table S2: Compositional parameters of each layer in chiplet-based heterogeneously integrated AI chip model with integrated vein-bionic fractal microchannel manifold structure, Table S3: Analysis of variance results of orthogonal experiment, Table S4: Training sample datasets (250), Text S1: Fluid mechanics, Text S2: Manifold pressure drop.
