# Supplementary material for: Research on Intelligent Thermal Optimization for Chiplet-Based Heterogeneously Integrated AI Chip Embedded with Leaf-Vein-Inspired Fractal Microchannels
Source: Materials (Basel). 2026 Feb 10;19(4):679. doi: 10.3390/ma19040679 (PMC12941633; doi:10.3390/ma19040679)
Supplement: Supplementary file 1 [file materials-19-00679-s001.zip › materials-4092104-supplementary.pdf]

Supporting information

Research on Intelligent thermal optimization for chiplet-based heterogeneously integrated AI Chip embedded with leaf-vein-Inspired fractal microchannels

Jie Wu [1], Yu Liang [1], Guibin Liu [1], Ruiyang Pang [1], Yi Teng [1], Chen Li [2], Xuetian Bao [2], Shi Lei [2] and Cai Zhikuang [1]

Supplementary Catalog

Figure S1. Workflow diagram for multi-parameter optimization of bionic fractal microchannel structure

Figure S2. Planar layouts of four microchannel structures

Figure S3. Meshing of embedded venation biomimetic fractal microchannel manifold structures

Table S1. Dimensions of 3D stacked structural model

Table S2. Compositional parameters of each layer in chiplet-based heterogeneously integrated AI chip model with integrated vein-bionic fractal microchannel manifold structure

Table S3. Analysis of variance results of orthogonal experiment

Figure S4. Schematic diagram of primary and auxiliary channels in microchannel-embedded body (the region delineated by blue dashed lines represents the main flow channel, while the light blue shaded area indicates the secondary flow channel)

Text S1. Fluid mechanics

Table S4. Training sample datasets (250)

Figure S5. Grid independence verification

Text S2. Manifold pressure drop

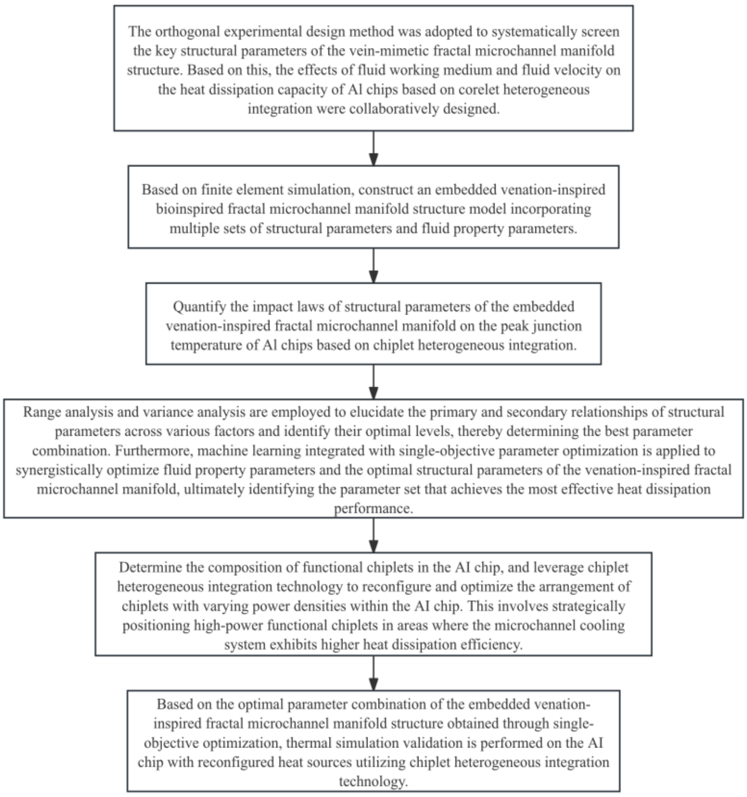

Figure S1. Workflow diagram for multi-parameter optimization of bionic fractal microchannel structure.

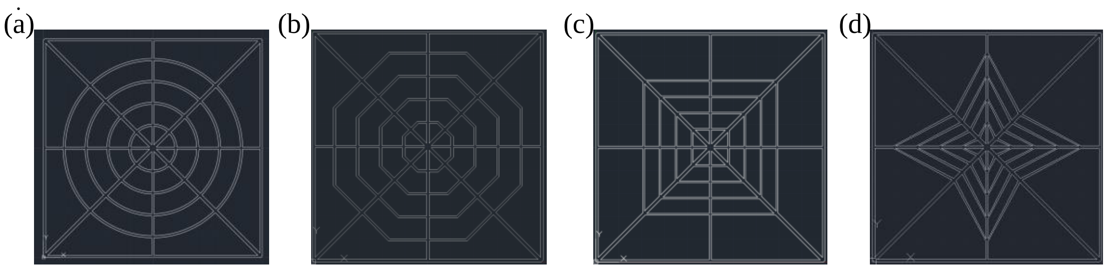

Microchannel planar structures: (a) circular; (b) octagonal; (c) quadrangular; (d) star-shaped.

Figure S2. Planar layouts of four microchannel structures

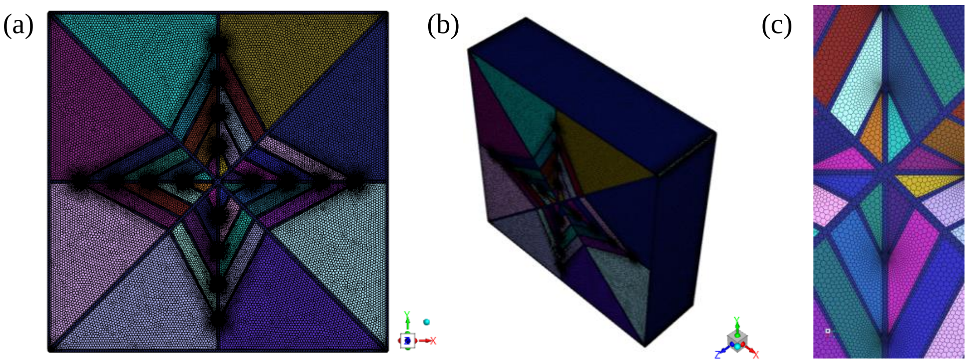

Mesh generation: (a) planar view; (b) 3D view; (c) partial enlarged view.

Figure S3. Meshing of embedded venation biomimetic fractal microchannel manifold structures

Table S1. Dimensions of 3D stacked structural model.

Name	Dimension	Materials
Interposer with TSV	120mm×120mm×6mm	Si
Chip	60mm×60mm×3mm	Si
Bump	60mm×60mm×3mm	60Sn40Pb
Solder ball	120mm×120mm×6mm	60Sn40Pb
PCB substrate	200mm×200mm×10mm	TU-752

Table S2. Compositional parameters of each layer in chiplet-based heterogeneously integrated AI chip model with integrated vein-bionic fractal microchannel manifold structure.

Composition	Title	Density /(kg·m-1)	Thermal conductivity /(W·m-1·K-1)	Specific heat capacity /(J·kg-1·K-1)
Solder ball/bump	60Sn40Pb	9000	50	150
Filler	epoxy resin	1300	0.5	750
PCB substrate	TU-752	1800	0.51	2000
Micro-solder joint	63Sn37Pb	8.4	50	183

Table S3. Analysis of variance results of orthogonal experiment.

Source of variance	Sum of squared deviations	Degrees of freedom	Root mean square	F-number	Critical value Fα	Significance
Element A	$\mathrm{SS_A}$	$\mathrm{d}f_A$	$\frac{\mathrm{SS_A}}{\mathrm{d}f_A}$	$\frac{\mathrm{MS_A}}{MS_e}$	(table look-up)	
Element B	$\mathrm{SS_B}$	$\mathrm{d}f_B$	$\frac{\mathrm{SS_B}}{\mathrm{d}f_B}$	$\frac{\mathrm{MS_B}}{MS_e}$	(table look-up)	
...	...	...	...	...		
Error e	$\mathrm{SS_T}$	$\mathrm{d}f_e$	$\frac{\mathrm{SS_e}}{\mathrm{d}f_e}$			
Summation	$\mathrm{SS_T}$	$\mathrm{d}f_T$	$\frac{\mathrm{SS_T}}{\mathrm{d}f_T}$			

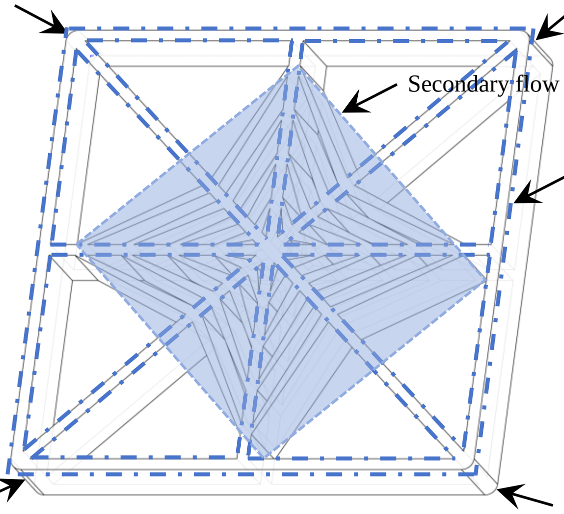

Figure S4. Schematic diagram of primary and auxiliary channels in microchannel-embedded body (the region delineated by blue dashed lines represents the main flow channel, while the light blue shaded area indicates the secondary flow channel).

Text S1: Fluid mechanics

The CFD simulation employed the ANSYS Fluent pressure-based solver, with the mass, momentum, energy equations, and the Realizable k-ε turbulence model configured as per the default settings.

Mass conservation equation:

$$\frac{\partial \rho}{\partial t} = \frac{\partial(\rho u_x)}{\partial x} + \frac{\partial(\rho u_y)}{\partial y} + \frac{\partial(\rho u_z)}{\partial z}$$

If the fluid is assumed to be an ideal fluid, i.e., incompressible with constant density, the above equation can be written as:

$$\frac{\partial u_x}{\partial x} + \frac{\partial u_y}{\partial y} + \frac{\partial u_z}{\partial z} = 0$$

In the equation, u_x、u_y、u_z are the linear displacement components in each direction of the Cartesian coordinate system, and ρ is the fluid density.

The above equation indicates that for a fluid with constant density, the total volume remains constant over time, signifying mass conservation.

Momentum conservation equations:

$$\frac{\partial(\rho u_x)}{\partial t} + \nabla \cdot (\rho u_x \vec{u}) = -\frac{\partial p}{\partial x} + \frac{\partial \tau_{xx}}{\partial x} + \frac{\partial \tau_{yx}}{\partial y} + \frac{\partial \tau_{zx}}{\partial z} + \rho a_x$$

$$\frac{\partial(\rho u_y)}{\partial t} + \nabla \cdot (\rho u_y \vec{u}) = -\frac{\partial p}{\partial x} + \frac{\partial \tau_{xy}}{\partial x} + \frac{\partial \tau_{yy}}{\partial y} + \frac{\partial \tau_{zy}}{\partial z} + \rho a_y$$

$$\frac{\partial(\rho u_z)}{\partial t} + \nabla \cdot (\rho u_z \vec{u}) = -\frac{\partial p}{\partial x} + \frac{\partial \tau_{xz}}{\partial x} + \frac{\partial \tau_{yz}}{\partial y} + \frac{\partial \tau_{zz}}{\partial z} + \rho a_z$$

In the equations, τ_{xx}、τ_{xy}、τ_{xz} represent the viscous force components in each direction of the Cartesian coordinate system; ρ is the mass density of the medium; a_x、a_y、a_z are the acceleration components in each direction of the Cartesian coordinate system.

According to the momentum theorem, the momentum of matter in an isolated system remains constant. Changes in

infinitesimal momentum arise from external forces, equaling the product of the sum of all external forces acting on the system and time.

Energy conservation equation:

$$\frac{\partial(\rho T)}{\partial t}+\frac{\partial(\rho uT)}{\partial x}+\frac{\partial(\rho vT)}{\partial y}+\frac{\partial(\rho wT)}{\partial z}=\frac{\partial}{\partial x}\frac{\lambda}{C_P}\frac{\partial T}{\partial x}+\frac{\partial}{\partial x}\frac{\lambda}{C_P}\frac{\partial T}{\partial x}+\frac{\partial}{\partial y}\frac{\lambda}{C_P}\frac{\partial T}{\partial y}+\frac{\partial}{\partial z}\frac{\lambda}{C_P}\frac{\partial T}{\partial z}+S_T$$

In the equation, λ is the thermal conductivity of the medium, and S_T represents the work carried out by the conversion of mechanical energy to thermal energy by the internal heat source within the fluid due to viscous effects. According to the first law of thermodynamics, in an isolated system, changes in energy of an infinitesimal element equal the total work carried out on the element by external forces (body forces and surface forces), plus the net heat flux entering the element.

The numerical calculations were completed using the Realizable k-ε turbulence model, where the turbulent kinetic energy k and the dissipation rate ε satisfy:

$$\frac{\partial(pk)}{\partial t}+\frac{\partial(pku_i)}{\partial x_i}=\frac{\partial}{\partial x_j}\left[\left(\mu+\frac{\mu_t}{\sigma_k}\right)\frac{\partial k}{\partial x_j}\right]+G_k-\rho\varepsilon$$

$$\frac{\partial(p\varepsilon)}{\partial t}+\frac{\partial(p\varepsilon u_i)}{\partial x_i}=\frac{\partial}{\partial x_j}\left[\left(\mu+\frac{\mu_t}{\sigma_\varepsilon}\right)\frac{\partial \varepsilon}{\partial x_j}\right]+\rho C_1 E\varepsilon-\rho C_2\frac{\varepsilon^2}{k+\sqrt{v\varepsilon}}$$

In the equation, σ_k=1.0， σ_ε=1.2， C_2=1.9， C_1=Max$\left(0.43,\frac{\eta}{\eta+5}\right)$,$\eta=\left(2E_{ij},E_{ij}\right)^{1/2}\frac{k}{\varepsilon}$, $E_{ij}=\frac{1}{2}\left(\frac{\partial u_i}{\partial x_j}+\frac{\partial u_j}{\partial x_i}\right)$.

where k is the turbulent kinetic energy; ε is the dissipation rate of turbulent kinetic energy; μ_t is the turbulent viscosity coefficient; ρ、μ、u、ν represent the fluid density, kinematic viscosity coefficient, flow velocity, and dynamic viscosity coefficient, respectively, G_k is the generation term of turbulent kinetic energy k caused by the mean velocity gradient; and σ_k and σ_ε are the Prandtl numbers corresponding to the turbulent kinetic energy k and dissipation rate ε, respectively.

Table S4. Training sample datasets (250).

Number of columns	C1	C2	C3	C4	C5	C6	C7
Number	A	B	C	D	E	F	Chip junction temperature (°C)
1	H2O	0.5	5	I	0.5	1	42.01
2	CH3COCH3	0.5	5	I	0.5	1	127.85
3	H2O	0.5	5	I	0.5	2	36.75
4	CH3COCH3	0.5	5	I	0.5	2	82.47
5	H2O	0.5	5	I	0.5	3	34.88
6	CH3COCH3	0.5	5	I	0.5	3	67.29
7	H2O	0.5	5	I	0.5	4	33.92

8	CH3COCH3	0.5	5	I	0.5	4	59.63
9	H2O	0.5	5	I	0.5	5	33.32
10	CH3COCH3	0.5	5	I	0.5	5	54.79
11	H2O	0.75	10	II	1	1	35.11
12	CH3COCH3	0.75	10	II	1	1	64.79
13	H2O	0.75	10	II	1	2	33.04
14	CH3COCH3	0.75	10	II	1	2	49.32
15	H2O	0.75	10	II	1	3	32.25
16	CH3COCH3	0.75	10	II	1	3	44.78
17	H2O	0.75	10	II	1	4	31.83
18	CH3COCH3	0.75	10	II	1	4	41.9
19	H2O	0.75	10	II	1	5	31.56
20	CH3COCH3	0.75	10	II	1	5	40.06
21	H2O	1	15	III	1.5	1	32.57
22	CH3COCH3	1	15	III	1.5	1	46.93
23	H2O	1	15	III	1.5	2	32
24	CH3COCH3	1	15	III	1.5	2	40.94
25	H2O	1	15	III	1.5	3	31.63
26	CH3COCH3	1	15	III	1.5	3	38.3
27	H2O	1	15	III	1.5	4	31.33
28	CH3COCH3	1	15	III	1.5	4	36.84
29	H2O	1	15	III	1.5	5	31.12
30	CH3COCH3	1	15	III	1.5	5	35.88
31	H2O	1.25	20	IV	2	1	34.5

32	CH3COCH3	1.25	20	IV	2	1	55.86
33	H2O	1.25	20	IV	2	2	33.21
34	CH3COCH3	1.25	20	IV	2	2	46.5
35	H2O	1.25	20	IV	2	3	32.7
36	CH3COCH3	1.25	20	IV	2	3	42.76
37	H2O	1.25	20	IV	2	4	32.4
38	CH3COCH3	1.25	20	IV	2	4	40.76
39	H2O	1.25	20	IV	2	5	32.17
40	CH3COCH3	1.25	20	IV	2	5	39.48
41	H2O	1.5	25	V	2.5	1	37.59
42	CH3COCH3	1.5	25	V	2.5	1	57.64
43	H2O	1.5	25	V	2.5	2	34.57
44	CH3COCH3	1.5	25	V	2.5	2	48.04
45	H2O	1.5	25	V	2.5	3	33.48
46	CH3COCH3	1.5	25	V	2.5	3	44.18
47	H2O	1.5	25	V	2.5	4	32.99
48	CH3COCH3	1.5	25	V	2.5	4	42
49	H2O	1.5	25	V	2.5	5	32.69
50	CH3COCH3	1.5	25	V	2.5	5	40.58
51	CH3COCH3	0.5	10	III	2	1	65.54
52	Coolant oil	0.5	10	III	2	1	41.08
53	CH3COCH3	0.5	10	III	2	2	50.03
54	Coolant oil	0.5	10	III	2	2	36.91
55	CH3COCH3	0.5	10	III	2	3	44.36

56	Coolant oil	0.5	10	III	2	3	35.34
57	CH3COCH3	0.5	10	III	2	4	41.35
58	Coolant oil	0.5	10	III	2	4	34.48
59	CH3COCH3	0.5	10	III	2	5	39.55
60	Coolant oil	0.5	10	III	2	5	33.93
61	CH3COCH3	0.75	15	IV	2.5	1	73.45
62	Coolant oil	0.75	15	IV	2.5	1	43.97
63	CH3COCH3	0.75	15	IV	2.5	2	54.95
64	Coolant oil	0.75	15	IV	2.5	2	38.8
65	CH3COCH3	0.75	15	IV	2.5	3	48.35
66	Coolant oil	0.75	15	IV	2.5	3	36.97
67	CH3COCH3	0.75	15	IV	2.5	4	44.78
68	Coolant oil	0.75	15	IV	2.5	4	35.98
69	CH3COCH3	0.75	15	IV	2.5	5	42.57
70	Coolant oil	0.75	15	IV	2.5	5	35.36
71	CH3COCH3	1	20	V	0.5	1	78.95
72	Coolant oil	1	20	V	0.5	1	48
73	CH3COCH3	1	20	V	0.5	2	60.1
74	Coolant oil	1	20	V	0.5	2	43.14
75	CH3COCH3	1	20	V	0.5	3	52.6
76	Coolant oil	1	20	V	0.5	3	41.14
77	CH3COCH3	1	20	V	0.5	4	48.43
78	Coolant oil	1	20	V	0.5	4	39.35
79	CH3COCH3	1	20	V	0.5	5	45.76

80	Coolant oil	1	20	V	0.5	5	38.76
81	CH3COCH3	1.25	25	I	1	1	42.08
82	Coolant oil	1.25	25	I	1	1	35.6
83	CH3COCH3	1.25	25	I	1	2	38.04
84	Coolant oil	1.25	25	I	1	2	34.28
85	CH3COCH3	1.25	25	I	1	3	36.32
86	Coolant oil	1.25	25	I	1	3	33.77
87	CH3COCH3	1.25	25	I	1	4	35.29
88	Coolant oil	1.25	25	I	1	4	33.49
89	CH3COCH3	1.25	25	I	1	5	34.6
90	Coolant oil	1.25	25	I	1	5	33.29
91	CH3COCH3	1.5	5	II	1.5	1	67.47
92	Coolant oil	1.5	5	II	1.5	1	47.01
93	CH3COCH3	1.5	5	II	1.5	2	52.37
94	Coolant oil	1.5	5	II	1.5	2	42.1
95	CH3COCH3	1.5	5	II	1.5	3	46.69
96	Coolant oil	1.5	5	II	1.5	3	40.28
97	CH3COCH3	1.5	5	II	1.5	4	43.61
98	Coolant oil	1.5	5	II	1.5	4	39.26
99	CH3COCH3	1.5	5	II	1.5	5	41.64
100	Coolant oil	1.5	5	II	1.5	5	39.7
101	Coolant oil	0.5	15	V	1	1	60.12
102	50%C2H6O2	0.5	15	V	1	1	46.49
103	Coolant oil	0.5	15	V	1	2	48.02

104	50%C2H6O2	0.5	15	V	1	2	39.63
105	Coolant oil	0.5	15	V	1	3	43.53
106	50%C2H6O2	0.5	15	V	1	3	37.14
107	Coolant oil	0.5	15	V	1	4	41.17
108	50%C2H6O2	0.5	15	V	1	4	35.81
109	Coolant oil	0.5	15	V	1	5	39.7
110	50%C2H6O2	0.5	15	V	1	5	34.96
111	Coolant oil	0.75	20	I	1.5	1	36.95
112	50%C2H6O2	0.75	20	I	1.5	1	33.65
113	Coolant oil	0.75	20	I	1.5	2	34.66
114	50%C2H6O2	0.75	20	I	1.5	2	32.41
115	Coolant oil	0.75	20	I	1.5	3	33.86
116	50%C2H6O2	0.75	20	I	1.5	3	31.94
117	Coolant oil	0.75	20	I	1.5	4	33.39
118	50%C2H6O2	0.75	20	I	1.5	4	31.7
119	Coolant oil	0.75	20	I	1.5	5	33.07
120	50%C2H6O2	0.75	20	I	1.5	5	31.53
121	Coolant oil	1	25	II	2	1	36.32
122	50%C2H6O2	1	25	II	2	1	33.73
123	Coolant oil	1	25	II	2	2	34.66
124	50%C2H6O2	1	25	II	2	2	32.67
125	Coolant oil	1	25	II	2	3	33.57
126	50%C2H6O2	1	25	II	2	3	32.27
127	Coolant oil	1	25	II	2	4	33.14

128	50%C2H6O2	1	25	II	2	4	32.04
129	Coolant oil	1	25	II	2	5	32.93
130	50%C2H6O2	1	25	II	2	5	31.85
131	Coolant oil	1.25	5	III	2.5	1	45.79
132	50%C2H6O2	1.25	5	III	2.5	1	37.37
133	Coolant oil	1.25	5	III	2.5	2	41.84
134	50%C2H6O2	1.25	5	III	2.5	2	35.46
135	Coolant oil	1.25	5	III	2.5	3	40.31
136	50%C2H6O2	1.25	5	III	2.5	3	34.53
137	Coolant oil	1.25	5	III	2.5	4	39.16
138	50%C2H6O2	1.25	5	III	2.5	4	34.03
139	Coolant oil	1.25	5	III	2.5	5	38.25
140	50%C2H6O2	1.25	5	III	2.5	5	33.8
141	Coolant oil	1.5	10	IV	0.5	1	46.05
142	50%C2H6O2	1.5	10	IV	0.5	1	38.18
143	Coolant oil	1.5	10	IV	0.5	2	40.89
144	50%C2H6O2	1.5	10	IV	0.5	2	35.71
145	Coolant oil	1.5	10	IV	0.5	3	38.9
146	50%C2H6O2	1.5	10	IV	0.5	3	34.54
147	Coolant oil	1.5	10	IV	0.5	4	37.92
148	50%C2H6O2	1.5	10	IV	0.5	4	33.91
149	Coolant oil	1.5	10	IV	0.5	5	37.31
150	50%C2H6O2	1.5	10	IV	0.5	5	33.55
151	50%C2H6O2	0.5	20	II	2.5	1	35.02

152	4%AI2O3 aqueous solution	0.5	20	II	2.5	1	34.1
153	50%C2H6O2	0.5	20	II	2.5	2	33.32
154	4%AI2O3 aqueous solution	0.5	20	II	2.5	2	32.85
155	50%C2H6O2	0.5	20	II	2.5	3	32.61
156	4%AI2O3 aqueous solution	0.5	20	II	2.5	3	32.31
157	50%C2H6O2	0.5	20	II	2.5	4	32.21
158	4%AI2O3 aqueous solution	0.5	20	II	2.5	4	31.98
159	50%C2H6O2	0.5	20	II	2.5	5	31.94
160	4%AI2O3 aqueous solution	0.5	20	II	2.5	5	31.75
161	50%C2H6O2	0.75	25	III	0.5	1	32.54
162	4%AI2O3 aqueous solution	0.75	25	III	0.5	1	31.92
163	50%C2H6O2	0.75	25	III	0.5	2	31.69
164	4%AI2O3 aqueous solution	0.75	25	III	0.5	2	31.26
165	50%C2H6O2	0.75	25	III	0.5	3	31.29
166	4%AI2O3 aqueous solution	0.75	25	III	0.5	3	31.04
167	50%C2H6O2	0.75	25	III	0.5	4	31.08
168	4%AI2O3 aqueous solution	0.75	25	III	0.5	4	30.91
169	50%C2H6O2	0.75	25	III	0.5	5	30.92
170	4%AI2O3 aqueous solution	0.75	25	III	0.5	5	30.81
171	50%C2H6O2	1	5	IV	1	1	46.71
172	4%AI2O3 aqueous solution	1	5	IV	1	1	43.52
173	50%C2H6O2	1	5	IV	1	2	40.64
174	4%AI2O3 aqueous solution	1	5	IV	1	2	38.48

175	50%C2H6O2	1	5	IV	1	3	38.54
176	4%AI2O3 aqueous solution	1	5	IV	1	3	36.64
177	50%C2H6O2	1	5	IV	1	4	37.36
178	4%AI2O3 aqueous solution	1	5	IV	1	4	35.62
179	50%C2H6O2	1	5	IV	1	5	36.64
180	4%AI2O3 aqueous solution	1	5	IV	1	5	34.94
181	50%C2H6O2	1.25	10	V	1.5	1	44.32
182	4%AI2O3 aqueous solution	1.25	10	V	1.5	1	41.76
183	50%C2H6O2	1.25	10	V	1.5	2	40.62
184	4%AI2O3 aqueous solution	1.25	10	V	1.5	2	37.55
185	50%C2H6O2	1.25	10	V	1.5	3	39.35
186	4%AI2O3 aqueous solution	1.25	10	V	1.5	3	35.91
187	50%C2H6O2	1.25	10	V	1.5	4	37.67
188	4%AI2O3 aqueous solution	1.25	10	V	1.5	4	34.94
189	50%C2H6O2	1.25	10	V	1.5	5	36.99
190	4%AI2O3 aqueous solution	1.25	10	V	1.5	5	34.32
191	50%C2H6O2	1.5	15	I	2	1	40.74
192	4%AI2O3 aqueous solution	1.5	15	I	2	1	33.57
193	50%C2H6O2	1.5	15	I	2	2	38.05
194	4%AI2O3 aqueous solution	1.5	15	I	2	2	35.08
195	50%C2H6O2	1.5	15	I	2	3	36.29
196	4%AI2O3 aqueous solution	1.5	15	I	2	3	32.88
197	50%C2H6O2	1.5	15	I	2	4	35.8

198	4%AI2O3 aqueous solution	1.5	15	I	2	4	33.3
199	50%C2H6O2	1.5	15	I	2	5	34.58
200	4%AI2O3 aqueous solution	1.5	15	I	2	5	33.02
201	4%AI2O3 aqueous solution	0.5	25	IV	1.5	1	35.3
202	H2O	0.5	25	IV	1.5	1	35.38
203	4%AI2O3 aqueous solution	0.5	25	IV	1.5	2	33.33
204	H2O	0.5	25	IV	1.5	2	33.48
205	4%AI2O3 aqueous solution	0.5	25	IV	1.5	3	32.6
206	H2O	0.5	25	IV	1.5	3	32.76
207	4%AI2O3 aqueous solution	0.5	25	IV	1.5	4	32.19
208	H2O	0.5	25	IV	1.5	4	32.37
209	4%AI2O3 aqueous solution	0.5	25	IV	1.5	5	31.91
210	H2O	0.5	25	IV	1.5	5	32.11
211	4%AI2O3 aqueous solution	0.75	5	V	2	1	57
212	H2O	0.75	5	V	2	1	54.4
213	4%AI2O3 aqueous solution	0.75	5	V	2	2	46.36
214	H2O	0.75	5	V	2	2	46.5
215	4%AI2O3 aqueous solution	0.75	5	V	2	3	42.36
216	H2O	0.75	5	V	2	3	44.17
217	4%AI2O3 aqueous solution	0.75	5	V	2	4	40.25
218	H2O	0.75	5	V	2	4	42.52
219	4%AI2O3 aqueous solution	0.75	5	V	2	5	38.9
220	H2O	0.75	5	V	2	5	41.22

221	4%AI2O3 aqueous solution	1	10	I	2.5	1	34.34
222	H2O	1	10	I	2.5	1	34.36
223	4%AI2O3 aqueous solution	1	10	I	2.5	2	32.86
224	H2O	1	10	I	2.5	2	33.35
225	4%AI2O3 aqueous solution	1	10	I	2.5	3	32.31
226	H2O	1	10	I	2.5	3	33.19
227	4%AI2O3 aqueous solution	1	10	I	2.5	4	32.02
228	H2O	1	10	I	2.5	4	32.19
229	4%AI2O3 aqueous solution	1	10	I	2.5	5	31.83
230	H2O	1	10	I	2.5	5	31.83
231	4%AI2O3 aqueous solution	1.25	15	II	0.5	1	33.4
232	H2O	1.25	15	II	0.5	1	33.42
233	4%AI2O3 aqueous solution	1.25	15	II	0.5	2	32.29
234	H2O	1.25	15	II	0.5	2	33.19
235	4%AI2O3 aqueous solution	1.25	15	II	0.5	3	31.83
236	H2O	1.25	15	II	0.5	3	32.13
237	4%AI2O3 aqueous solution	1.25	15	II	0.5	4	31.58
238	H2O	1.25	15	II	0.5	4	31.9
239	4%AI2O3 aqueous solution	1.25	15	II	0.5	5	31.41
240	H2O	1.25	15	II	0.5	5	31.67
241	4%AI2O3 aqueous solution	1.5	20	III	1	1	32.01
242	H2O	1.5	20	III	1	1	32.84
243	4%AI2O3 aqueous solution	1.5	20	III	1	2	31.58

244	H2O	1.5	20	III	1	2	31.91
245	4%AI2O3 aqueous solution	1.5	20	III	1	3	31.38
246	H2O	1.5	20	III	1	3	31.52
247	4%AI2O3 aqueous solution	1.5	20	III	1	4	31.26
248	H2O	1.5	20	III	1	4	31.26
249	4%AI2O3 aqueous solution	1.5	20	III	1	5	31.17
250	H2O	1.5	20	III	1	5	31.06

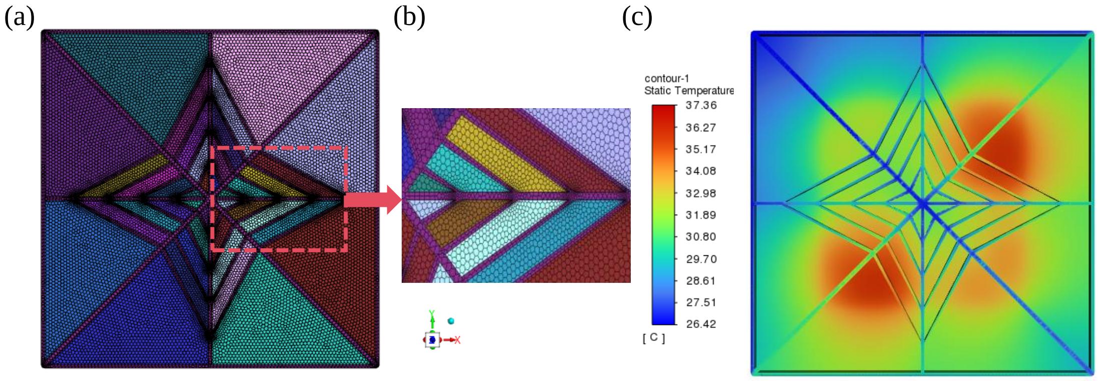

I. Coarse grid: (a) planar view; (b) partial enlarged view; (c) chip junction temperature simulation results.

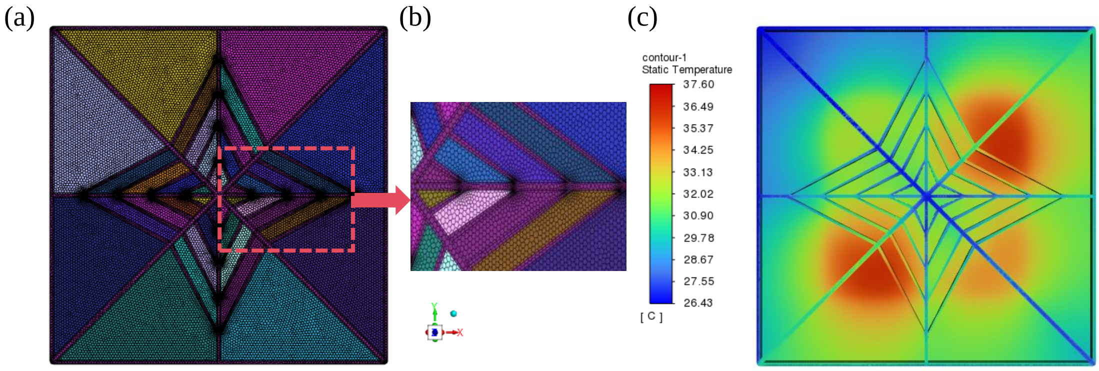

II. Medium grid: (a) planar view; (b) partial enlarged view; (c) chip junction temperature simulation results.

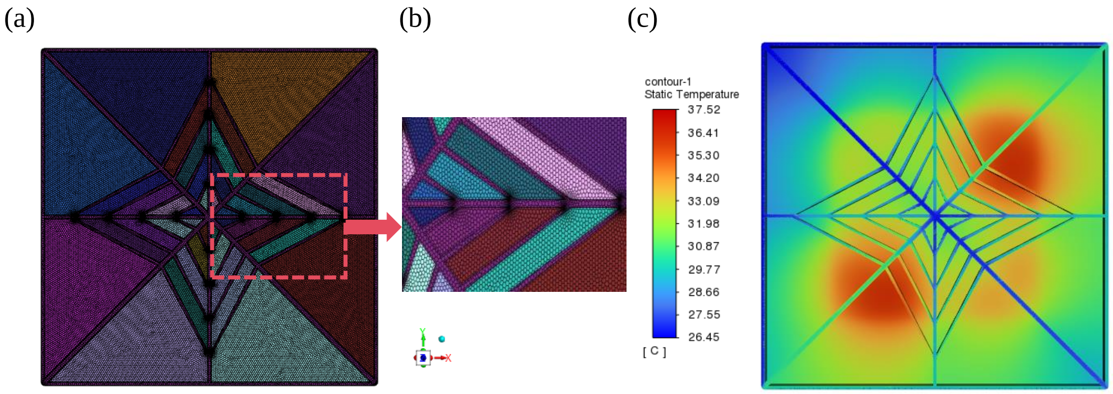

III. Fine grid: (a) planar view; (b) partial enlarged view; (c) chip junction temperature simulation results.

Figure S5. Grid independence verification.

Text S2. Manifold pressure drop.

The pressure drops at the inlet and outlet are:

$$\Delta P_{in} = K_{in} \times \frac{\rho u_{in}}{2} = 0.12 \times \frac{1073.4 \times 4}{2} = 257.62\text{Pa}$$

$$\Delta P_{out} = K_{out} \times \frac{\rho u_{out}}{2} = \frac{1073.4 \times 4}{2} = 2146.8\text{Pa}$$

where ρ is the mass density of the coolant, K_{in} is the loss coefficient for the sudden contraction inlet, and $K_{out}$is the loss coefficient for the sudden expansion outlet. Typically, for a right-angle inlet, K_{in} = 0.5. For a rounded inlet, K_{in} = 0.03. For a micro-rounded inlet, K_{in} = 0.12. K_{out} is the easiest to determine and approximately equal to 1.

The frictional pressure drop in the microchannel is:

$$\Delta P_c = f \frac{\rho u}{2}\left(\frac{L}{D_h}\right)$$

where

$$f = \frac{K(\infty)}{4[L/(D_h \times Re)]} + \frac{96/Re}{\left[1 + \frac{1}{\alpha}\right]^2 \left[1 - \frac{192}{\pi^5 \alpha} \sum_{n=1,3,5}^{\infty} \left(\frac{\tanh(n\pi\alpha/2)}{n^5}\right)\right]}$$

$$K(\infty) = 0.6796 + 1.2197\alpha + 3.3089\alpha^2 - 9.5921\alpha^3 + 8.9089\alpha^4 - 2.9959\alpha^5$$

$$D_h = \frac{4S}{C} = \frac{4 \times 27}{21.9017} \approx 4.93\text{mm} = 0.00493\text{m}$$

$$Re = \frac{\rho u D_h}{\mu} = \frac{1073.4 \times 4 \times 0.00493}{0.00394} \approx 5372.45$$

where f is the friction coefficient, ρ is the fluid density, L is the flow path length, u is the velocity magnitude, and D_h is the hydraulic diameter. $K(\infty)$ is the constant Hagenbach factor, Re is the Reynolds number, and μ is the viscosity of the coolant. The constant Hagenbach factor is a function of the aspect ratio α ($0 < \alpha < 1$). S is the channel area, and C is the channel perimeter. It can be seen from the equation that the frictional pressure drop is proportional to the

friction coefficient f, flow length L, and velocity magnitude u, and inversely proportional to the hydraulic diameter D_h.

Aspect ratio of the microchannel:

$$\alpha = \frac{0.75}{25} = 0.03\text{，obtain } K(\infty) \approx 0.72\text{，} f = 217.68\text{，} \quad \Delta P_c = 2076.07\text{Pa}$$

Total pressure drop:

$$\Delta P = \Delta P_{in} + \Delta P_{out} + \Delta P_c = 257.62 + 2146.8 + 2076.07 = 4480.49\text{Pa}$$
